# Target and Nontarget Screening of Organic Chemicals and Metals in Recycled Plastic Materials

**DOI:** 10.1021/acs.est.2c07254

**Published:** 2023-02-14

**Authors:** Leah Chibwe, Amila O. De Silva, Christine Spencer, Camilla F. Teixera, Mary Williamson, Xiaowa Wang, Derek C. G. Muir

**Affiliations:** † Aquatic Contaminants Research Division, Environment Climate Change Canada, Burlington, Ontario L7S 1A1, Canada; ‡ Institute for Environmental Change and Society, University of Regina, Regina, Saskatchewan S4S 0A2, Canada

**Keywords:** recycling, plastics, contaminants, circular economy, nontargeted analysis

## Abstract

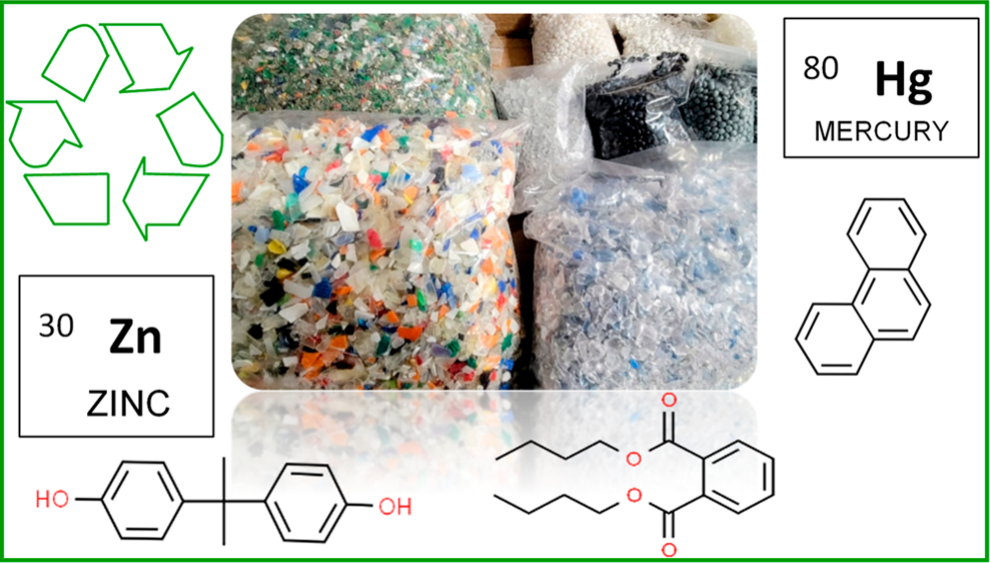

Increased demand for recycling plastic has prompted concerns regarding potential introduction of hazardous chemicals into recycled goods. We present a broad screening of chemicals in 21 plastic flake and pellet samples from Canadian recycling companies. From target analysis, the organophosphorus ester flame retardants and plasticizers exhibited the highest detection frequencies (DFs) (5–100%) and concentrations (<DL-4,700 ng/g), followed by brominated/chlorinated flame retardants (<DL-2,150 ng/g, 5–76% DFs). The perfluoroalkyl acids were least detected at the lowest concentrations (<0.01–0.70 ng/g, 5–19% DFs). Using nontargeted analysis, 217 chemicals were identified as Level 1 (authentic standard) or 2 (library match), with estimated individual concentrations up to 1030 ng/g (highest: 2-hexyl hydroxy benzoate, 100% DF). Total (Σ60) element concentrations were between 0.005 and 2,980 mg/kg, with highest concentrations for calcium (2,980 mg/kg), sodium (617 mg/kg), and iron (156 mg/kg). Collectively >280 chemicals were detected in recycled plastic pellets and flakes, suggesting potential incorporation into recycled goods. Individual concentrations indicate unintentional trace contamination following European Union threshold limits for recycled granules (500 mg/kg) and waste plastic flakes (1,000 mg/kg), although do not reflect toxicological thresholds, if any. Our study highlights that while recycling addresses sustainability goals, additional screening of goods and products made from recycled plastics is needed to fully document potentially hazardous chemicals and exposure.

## Abstract 2

>280 chemicals were detected in recycled flakes and pellets. Although recycling is intended to address sustainability goals, this study highlights the need for additional screening to fully document potential hazardous chemicals and exposure.

## Introduction

Worldwide plastic production currently surpasses 350 million tonnes per year and is projected to continue increasing.^1^ Policies to mitigate the effects of plastic pollution, such as the elimination of single use plastics,^2^ have been proposed. There is also growing demand for reusable and recycled plastics. Herein, plastic goods or products are reprocessed and recovered either for their original or alternate use, contributing toward the more sustainable “Circular Economy Model.”^3,4^

Presently, between 5 and 10% of plastics produced globally is recycled.^5^ In Canada, approximately 2,400 kilotons of postconsumer and commercial plastic were discarded with only 9% collected for recycling in 2016.^6,7^ These low recycling rates have prompted programs and initiatives that advocate for the increased use of recycled content and plastics.^8,9^ For example, the European Union (EU) has set recycling targets for plastic packaging at 50% and 55% by 2025 and 2030, respectively,^1^ while the American Chemistry Council Plastic Division aims that plastic packaging be 100% recyclable or reusable by 2040.^10^

This demand for increased recycling, however, draws concerns regarding the potential introduction of hazardous chemicals into recycled plastic products.^3,11,12^ Plastics contain many chemicals, including intentionally added substances (IAS) such as flame retardants, plasticizers, stabilizers, and antimicrobial agents—added during production to impart certain properties, such as versatility and stability.^9,13,14^ Other lesser-known chemicals are the nonauthorized nonintentionally added substances (NIAS).^9,13,14^ These substances can come into contact with plastics during their use, waste collection, sorting, and management, or be formed as byproducts during recycling processes.^15,16^ Legacy or phased-out chemicals, such as some phthalates that have been banned in plastic toys,^17^ can also resurface in recycled content from mixing with older products still in circulation or imported goods.^9,18,19^

Evidence for possible contamination of recycled plastic products and goods continues to grow. For example, flame retardants, plasticizers, and biocides were identified in recycled consumer products purchased from retailers in Texas, United States, including paper products, children’s toys, and food contact materials.^12^ Polybrominated biphenyls (PBBs) were detected in hard plastic toys purchased from various markets in China.^20^ The authors speculated the toys were likely manufactured from recycled plastics containing these halogenated compounds, as PBBs were banned in the 1970s in the United States and no records for their production exist in China.^20^ Halogenated flame retardants, including polybrominated diphenyl ethers, were also detected at concentrations up to 170,000 ng/g in recycled plastic materials, manufactured in China and purchased online.^21^ In The Netherlands, tetrabromobisphenol A and other flame retardants were reported at concentrations above 500 μg/g in products, such as toys and carpets, containing recycled content.^3^

There is still limited data on the composition and levels of potentially hazardous substances in recycled materials. For this reason, we provide a broad screening of chemicals in various plastic polymers obtained from Canadian recycling companies. Since previous studies predominantly address recycled consumer goods,^12,20,21^ our focus was on the analysis of plastic flakes and pellets—both intended for use in the production of recycled goods. Specifically, we used targeted gas (GC) and liquid chromatographic (LC) methods to analyze for brominated/chlorinated flame retardants (Br/Cl-FRs), organophosphorus ester flame retardants and plasticizers (OPEs), and perfluoroalkyl acids (PFAAs). We also applied nontargeted analysis (NTA) using LC high-resolution Orbitrap mass spectrometry (LC-HRMS) and comprehensive two-dimensional gas chromatography MS (GC×GC-ToFMS) to screen for a wide range of unknown organic compounds. Finally, we used inductively coupled plasma–mass spectrometry (ICP-MS) to obtain a multielement profile of the samples. Our results contribute knowledge to the issue of possible contamination of recycled goods and are of value to the broad scientific community, including regulators and the recycling industry.

## Materials and Methods

### Solvents and Chemicals

Organic solvents methanol (MeOH) and acetonitrile (ACN) were HPLC grade and purchased from EMD chemicals (Oakville, ON, Canada). HPLC grade water was purchased from Fisher Chemicals (Toronto, ON, Canada). All chemical standards were >96% purity and were purchased from Sigma-Aldrich (Oakville, ON, Canada), Toronto Research Chemicals (Toronto, ON, Canada), and Wellington Laboratories (Guelph, ON, Canada)

### Samples

Samples (*N* = 21) were obtained from five Canadian plastics recycling companies based in the provinces of Ontario and British Columbia and consisted of low-density polyethylene (LDPE), high-density polyethylene (HDPE), polypropylene (PP), polyethylene terephthalate (PET), wet fines (WF, i.e., raw product from cleaning and grinding PET/HDPE/PP polymers), high impact polystyrene (HIPS), and polyethylene (PE). All samples were dry solid materials, mainly flakes (cleaned and chipped original recycled materials) and pellets (produced by heating and extrusion of flakes) (Table 1). Samples were shipped in polyethylene Ziplock type bags and after receipt were labeled with unique sample numbers.

**Table 1:** Sample Descriptions and Codes of the Recycled Plastic Samples Studied

Code	Sample	Description
LDPE #1	Low density polyethylene-1	Gray pellets
LDPE #2	Low density polyethylene-2	Gray pellets
LLDPE #1	Linear low-density polyethylene G1 natural color film (finished)	White pellets
LLDPE #2	Linear low-density polyethylene G3 NAT REPRO	White pellets
LLDPE #3	Linear low-density polyethylene G1 NAT REPRO	White pellets
HDPE #1	High density polyethylene	Gray pellets
HDPE #2	High density polyethylene Pellets Gr	Gray pellets
PP #1	Polypropylene	Gray pellets
PP #2	Polypropylene Cap Flakes	Colored flakes
PP #3	Polypropylene Cap Flakes	Gray pellets
PP #4	Polypropylene REP	Black pellets
PP #5	Polypropylene REP	White pellets
PET #1	Polyethylene tetraphthalate Flakes (unwashed)	Colored flakes
PET #2	polyethylene tetraphthalate Flakes	Clear flakes
PET #3	polyethylene tetraphthalate REG	Clear flakes
WF #1	Wet Fines-1	White flakes
WF #2	Wet Fines-2	White flakes
WF #3	Wet Fines-3	White flakes
HIPS	High impact polystyrene NAT pellets REPRO	White pellets
PE #1	Polyethylene REVITAL-1	Gray pellets
PE #2	Polyethylene REVITAL-2	Gray pellets

### Sample Extraction

A nonspecific extraction method was implemented to process the plastic samples. This was based on methodology used by the USEPA for screening neutral or semipolar organic chemicals in consumer plastics.^22^ Briefly, 20 mL of dichloromethane (DCM) was added to 5 g of plastic material in a 40 mL-amber glass bottle (6% diethyl ether/hexane was used for the HIPS sample as the plastic dissolved in DCM). The extracts were evaporated to near dryness and taken up in 1 mL of hexane for GC analysis and in methanol for LC analysis. Labeled standards, phenanthrene-*d10* and chrysene-*d12*, were added to the extracts prior to extraction for GC×GC-ToFMS analysis. Labeled standards tris(2-tris(1-chloro-2-propyl) phosphate-*d18* (*d18* TCPP), sodium perfluoro-1-[^13^C_8_]octanesulfonate (M8PFOS), and perfluoro-*n*-[1,2-^13^C_2_]tetradecanoic acid (M2PFTeDA) were used as internal standards prior to LCMS analysis. Standards ^13^C-mirex and native BDE-71 and deuterated tris(2-chloroisopropyl) phosphate (*d18*-TCIPP) were added to the extracts prior to analysis for halogenated flame retardants and OPEs, respectively.

The extraction of PFAAs was carried out separately following previous methodology with slight modifications.^23^ Briefly, 5 g of plastic material was shaken in acetonitrile for 30 min, centrifuged, and extracted using methanol on carbon solid phase extraction columns. The extracts were then evaporated to near dryness and reconstituted to 1 mL in a 50:50 solution of methanol:water. Isotopically labeled (^13^C) internal standards of PFAAs (C4–C14 perfluorocarboxylates (PFCAs); C4–C12 perfluoroalkyl sulfonates (PFSAs); Wellington Laboratories, Guelph ON) were added prior to extraction. Detailed procedures for all methods are provided in the Supporting Information.

### GC-ECNI-MS

Extracts in hexane were analyzed for halogenated flame retardants (Br/Cl-FRs), including fifty-one PBDEs, using gas chromatography negative ion mass spectrometry (GC-ECNI-MS) (Table S1). The Br/Cl-FRs were determined using HP-5MS (Agilent Technologies, Inc., U.S.A.) or RTX-1614 (Restek Corporation, U.S.A.) capillary columns. Co-eluting congeners, including BDE-17/25, BDE-28/33, and BDE-138/166 were quantified together. PBDEs were monitored at *m*/*z* 79 and 81 and quantified using external standard calibration. Other Br/Cl-FRs were monitored by characteristic fragments (Table S1). Gas chromatographic oven temperature and mass spectral conditions are given in Table S2.

### LC/MS/MS

The samples were analyzed for PFAAs using negative electrospray ionization (ESI) tandem mass spectrometry (Table S1). Analytes were detected using an API 4000 Q Trap (Applied Biosystems, Carlsbad, CA) after chromatographic separation with an Agilent 1100 LC (injection volume = 40 μL, flow rate = 300 μL/min). Chromatography was performed using an ACE C18 column (50 mm × 2.1 mm, 3 μm particle size, Advanced Chromatography Technologies Ltd., Aberdeen, U.K.), preceded by a C18 guard column (4.0 × 2.0 mm, Phenomenex, Torrance, CA), and the column oven was set to 30 °C. Samples were quantified with a six-point calibration curve using the isotopic dilution method.

Samples were analyzed for OPEs using positive ESI (Table S1). Analytes were detected using an ultrahigh performance LC/MS/MS) consisting of Water XEVO TQS triple quadrupole MS coupled to a Water Acquity LC. Separation was performed using an Acquity UPLC BEH C_18_ column (Waters, 1.7 μm, 2.1 × 100 mm^2^) in a 60 °C thermostated compartment using 0.1% formic acid in water (A) and 0.1% formic acid in methanol (B) gradient.

### GC×GC-ToFMS

Analysis was conducted in electron ionization mode using a GC×GC/ToF-MS Pegasus 4D (LECO, St. Joseph, MI) equipped with an Agilent 7890B gas chromatograph (Palo Alto, CA). The columns used were the DB-1 MS UI (25 m × 0.25 mm × 0.25 μm; Agilent Technologies) and Rxi-17 Sil MS column (1.2 m × 0.25 mm × 0.25 μm; Restek) in the first and second dimension, respectively (Table S3).

### LC-HRMS

Chemical analysis was performed on a Thermo Vanquish ultrahigh-performance liquid chromatograph coupled to a Q Exactive Focus Orbitrap MS (Thermo Fisher Scientific, Mississauga, ON), using an Acquity UPLC BEH C_18_ column (2.1 mm × 50 mm, 1.7 μm particle size (Waters, Milford, MA, U.S.A.) for chromatographic separation. Further details are provided in the Supporting Information.

### Nontarget Screening and Identification

NTA was conducted using GC×GC-ToFMS and LC-HRMS to detect a broad range of nonpolar to polar analytes. GC×GC-ToFMS raw data was processed using the LECO ChromaToF v.4.50.8 software and included peak deconvolution and background subtraction. LC-HRMS raw data was processed using the Thermo Scientific Compound Discoverer software. Peak evaluation included retention time alignment, unknown compound detection, and elemental composition prediction (Table S4).

Priority for identification was on features (1) with matches in spectral libraries/databases, (2) MS^2^ fragmentation (LC) or adequate fragmentation (GC), and (3) peak areas at least 6× the area in blanks, that (4) were chromatographically resolved. As a result, peaks were identified as either Level 1: retention time (RT) and mass spectral match with authentic standard (GC and LC) or Level 2: >75% mzCloud database match (https://www.mzcloud.org/), Δmass < 5 ppm, isotope profile fit with theoretical >70% (LC), and NIST 11 EI mass spectral library >85% and isotope/fragmentation profile fit (GC).^24^ Additionally, other antioxidants, including octadecyl 3-(3,5-di-*tert*-butyl-4-hydroxyphenyl) propionate (Irganox 1076) and its cinnate derivative, were tentatively identified as Level 2 in LC-HRMS using MS^2^ literature comparison (Figure S1).^25^

### ICP-MS

Sixty elements were analyzed using inductively coupled plasma–mass spectrometry (ICP-MS) by the Environment and Climate Change Canada National Laboratory for Environmental Testing (NLET) Method 02-2404 based on U.S. Environmental Protection Agency Method 200.8.^26^ Elements analyzed included rare earth elements and the 12 priority pollutants elements (PPEs) (antimony, arsenic, beryllium, cadmium, chromium, copper, lead, nickel, selenium, silver, thallium, and zinc) (Table S5).

#### Quality Assurance and Quality Control

Solvent, procedural blanks (solvent extracted alongside samples), and calibration check standards were analyzed for every 6–8 samples to test for potential contamination and carry over during analysis for all methods. Mass accuracy calibration for the LC-HRMS was continuously maintained using the Pierce LTQ Velos ESI positive and negative ion calibration solutions (Thermo Scientific).

#### Data and Statistical analysis

Two-way analysis of variance (ANOVA) was used for each of the target classes (i.e., ΣOPEs, ΣBFRs, and sum (Σ) elements and NTA compounds (relative abundance) to determine the effect of form (pellets, *n* = 14, and flakes, *n* = 7) and color (colored, *n* = 11, and white/natural, *n* = 10) on the distribution of chemicals in the plastic samples. R Studio was used for assumption checking and two-way ANOVA analysis using the “dpylr”, “ggpubr”, and “car” packages.

In NTA, for comparison between samples and as proxy for the abundance of the chemicals, normalized peak areas (peak area normalized to labeled internal standards) were used. Additionally, NTA peak concentrations were estimated using external calibration curves of authentic standards; some were also used to confirm unknowns (Table S6). In GC×GC-ToFMS, peak concentrations were estimated using the average response factors of the authentic standards.^27^ In LC-HRMS, concentrations were estimated using the authentic standards closely matched by retention time (RT) and/or log *K*_ow_, with the assumption that these two parameters would reflect the behavior of the unknowns during extraction and analysis (Table S6). The working range for each standard was determined, and the coefficient of determination (*R*^2^) was >0.99 (Table S6).

## Results and Discussion

### Organic Chemical Analysis

**Figure 1:**
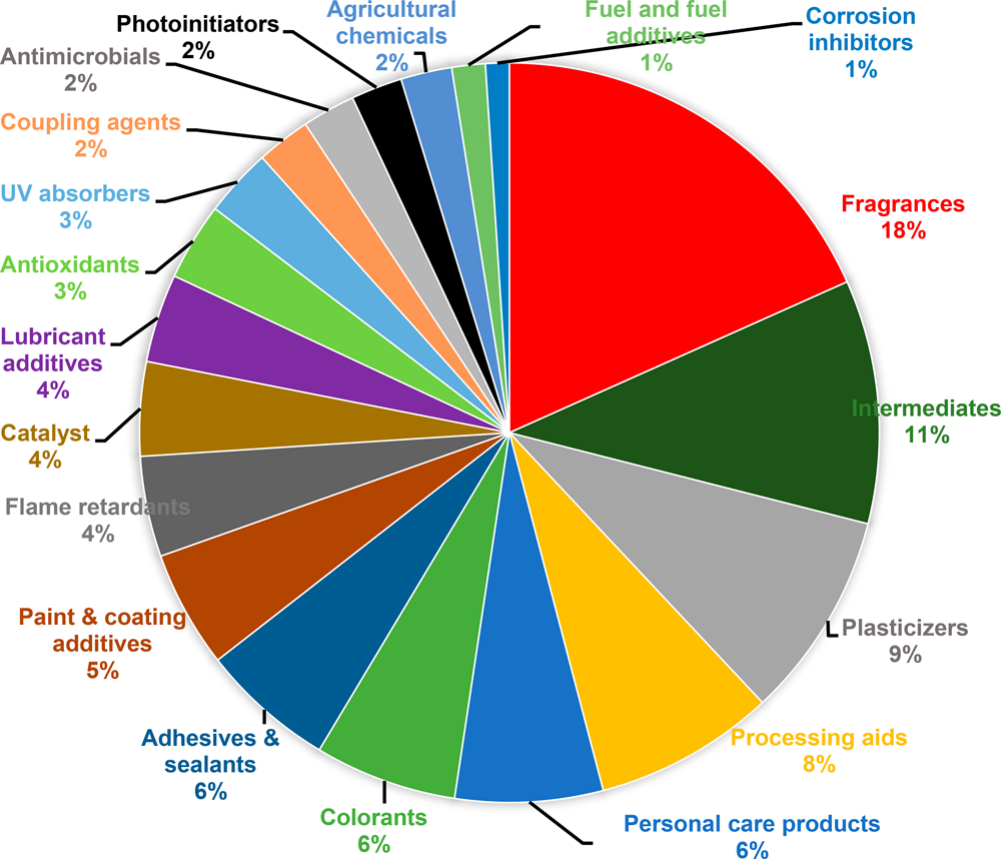
Pie chart that depicts detection frequency (%) of organic chemicals by industrial or industrial or functional use classification detected using target and nontarget analysis.

Collectively, organic chemicals were detected in recycled pellets and flakes using target and NTA. Using classifications from the PubChem “Industry Uses” section (https://pubchem.ncbi.nlm.nih.gov/) and the U.S. EPA functional use database (https://pasteur.epa.gov/uploads/495/functional_use_database.xlsx), chemicals were classified into several categories, including as plasticizers, fragrances, flame retardants, and antioxidants. A visual summary of chemical use classification based on detection frequencies (DFs) is provided in the pie chart in Figure 1. Fragrances (or perfumers/odor agents) were the most commonly detected. This included polycyclic aromatic compounds (PACs), such as alkylated naphthalenes and benzoic esters—predominantly detected in NTA by GC×GC/ToF-MS (Table S7). PACs have been reported in recycled consumer products^12^ and recycled LDPE pellets^27,28^ and have other industrial applications, including as solvents and intermediates.

Chemicals classified as intermediates and/or processing aids were the next frequently detected. This included several OPEs, such as tributyl phosphate (TNBP), triethyl phosphate (TEP), and 2-ethylhexyl diphenyl phosphate (EHDPP). Plasticizers, including several phthalates (also, classified by use as adhesives, sealants, and fillers) were also common (Table S7). Phthalates were present in over 85% of the samples, with the exception of dicyclohexyl phthalate (DCHP) (60% DF). Dibutyl phthalate (DBP), diethyl phthalate (DEP), diisobutyl phthalate (DIBP), and bis(2-hydroxyethyl) terephthalate (BHET) were the most abundant phthalates (by normalized peak area). These phthalates were also identified in consumer products made from recycled materials, including children’s toys and food contact materials,^12^ from LDPE recycled plastics,^29^ and in plastic bags containing at least 20% recycled PE plastic.^30^ Ionas et al. determined that phthalates, including DBP, BBP, and DEP, were present in most Belgian toys at levels not expected to impart beneficial properties^31^ and stipulated their presence was from cross-contamination during manufacture or recycling.^32^

**Figure 2:**
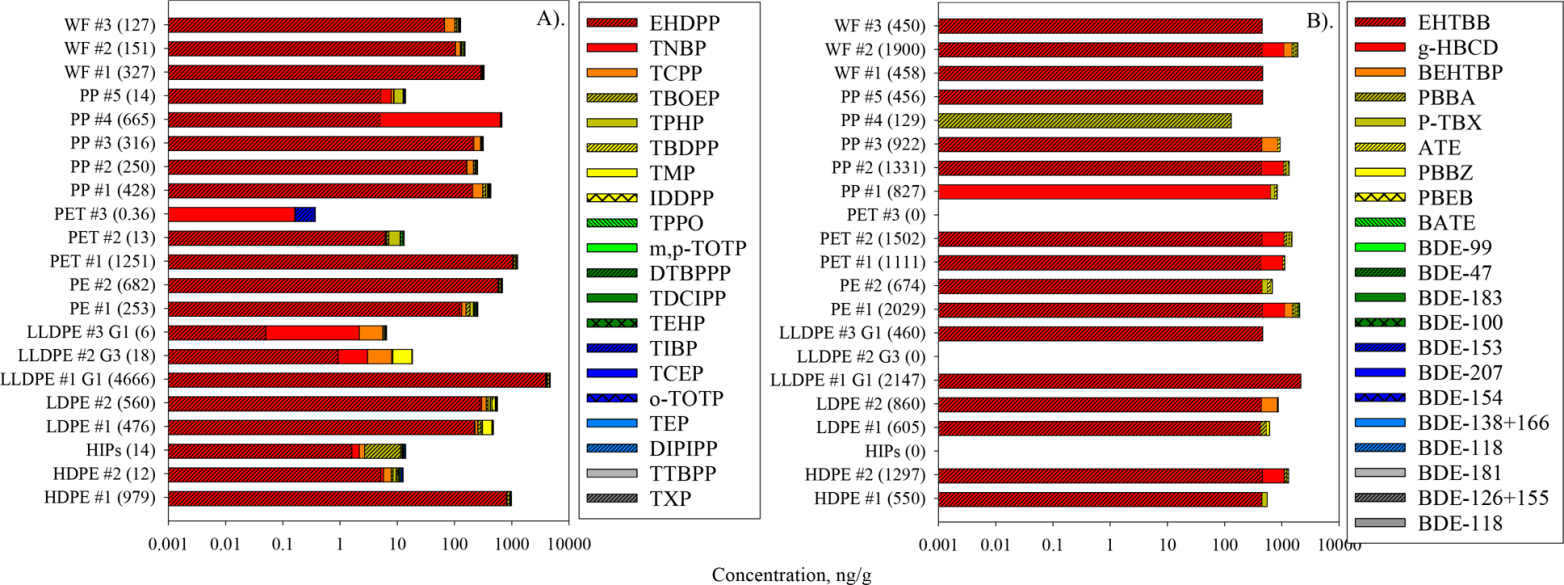
Chemical distribution (stacked bar, *x*-axis) of the targeted (A) organophosphate ester plasticizers and flame retardants and (B) halogenated flame retardants. Values in parentheses () represent total analyte concentration in ng/g in a given sample. Full names of plastic types and chemicals are provided in Tables 1 and S1, respectively.

Target analysis was used to determine the concentrations of specific OPEs, BFRs, and PFAAs. The highest concentration detected was for the OPE EHDPP (3,850 ng/g, 95% DF). Generally, the OPEs had the highest DFs (5–100%) and concentrations (ΣOPEs: <DL-4,700 ng/g) (Table S8, Figure 2A). Other abundant and/or commonly detected OPEs were TNBP (620 ng/g, 100% DF), (4-*tert*-butylphenyl) diphenyl phosphate (TBDPP), (280 ng/g, 67% DF) and trimethyl phosphate (TMP) (139 ng/g, 86% DF) (Table S8, Figure 2A). OPEs have applications as plasticizers in plastics and rubber and have been detected in consumer products, including curtains and LCD-TVs, at concentrations ranging from <0.0003 to 140,000 μg/g.^33^ Their predominance could be as a result of their increased production in recent years due to the global ban of certain brominated flame retardants.^34^

BFRs including polybrominated diphenyl ether (PBDEs) congeners were the next most prominent target class (ΣBFRs: <DL-2,150 ng/g) (Table S8, Figure 2B). The most abundant BFRs were 2-ethyl-1-hexyl 2,3,4,5-tetrabromobenzoate (EHTBB) (2,150 ng/g, 76% DF), γ-hexabromocyclododecane (g-HBCD) (650 ng/g, 33% DF), and bis(2-ethyl-1-hexyl) tetrabromophthalate (BEHTP) (406 ng/g, 19% DF) (Table S8, Figure 2B). Several PBDE congeners were detected in at least two samples (ΣPBDEs: <DL-32.1 ng/g, 5–48% DFs) (Table S8). Some PBDEs have been reported, though at comparably higher concentrations, in other related studies, including in recycled plastics from online retailers in China (range for Σ_10_PBDEs < LOD-7,500 ng/g),^21^ toys from a Belgian recycling park up to 143 × 10^6^ ng/g, mean: 4500 ng/g for Σ_10_BDEs), and various recycled plastic materials, including beverage cartons, from the Czech republic (median concentrations up to 4,000 ng/g for Σ_10_PBDEs congeners).^31,35^ The PFAAs were detected at the lowest concentrations and DFs (ΣPFAAs: <DL-0.693 ng/g, 5–19% DFs) (Table S8). PFAAs have been reported in various consumer products and household items at concentrations up to 78 ng/g.^36^ To the best of our knowledge, there is only one other study to have evaluated PFAAs in recycled materials, but it did not detect these chemicals in the household plastics sampled.^36^ The study proposed that since PFAAs are generally applied as surficial treatments, they are most likely to be released into the environment.^36^ Currently, because data is scarce, more research would be needed to characterize and understand levels and any possible contamination of recycled materials by PFAAs.

**Figure 3:**
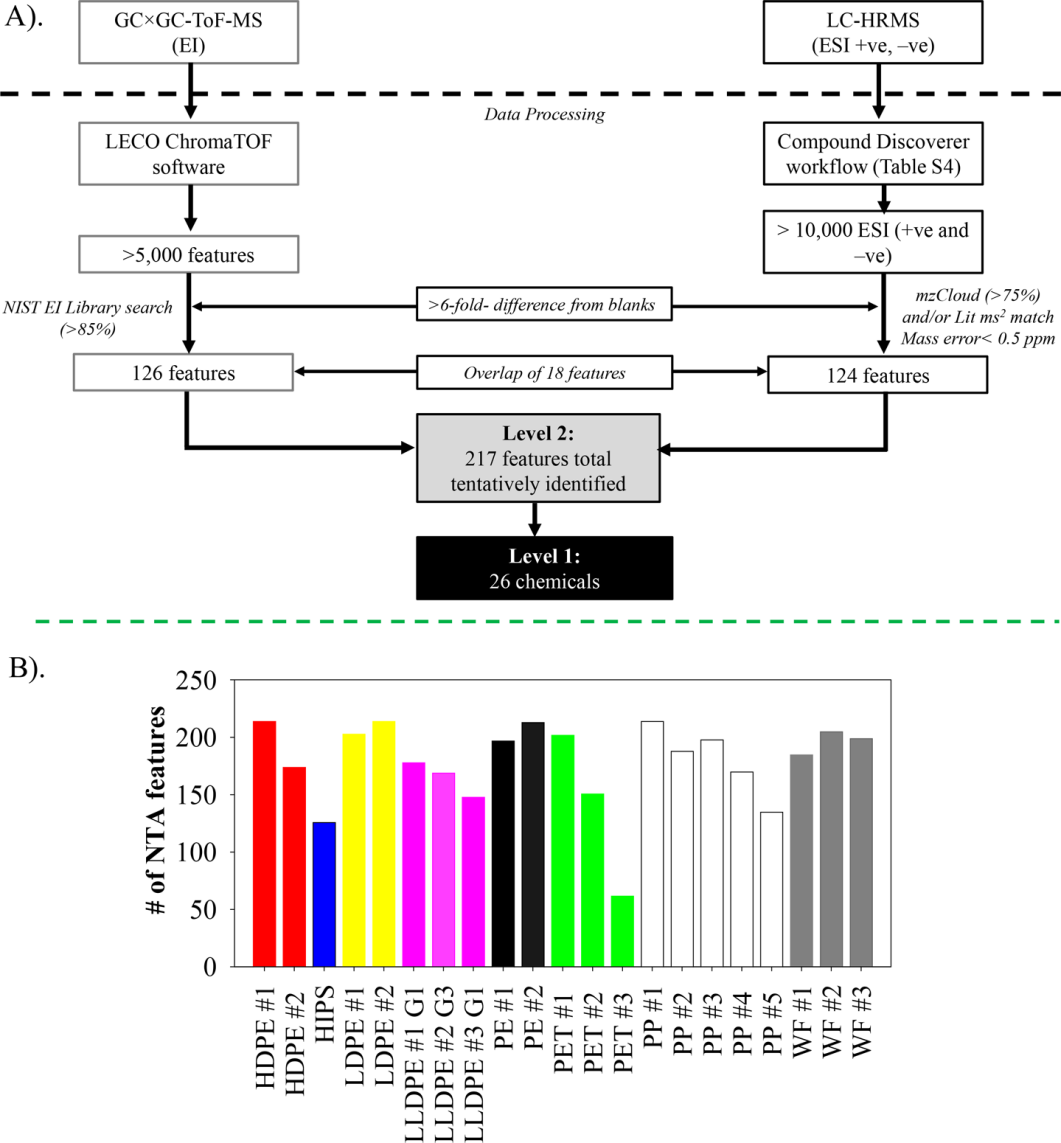
(A) Nontargeted analysis data processing workflow and summary of features identified using liquid chromatography high-resolution Orbitrap mass spectrometry (LC-HRMS) and comprehensive two-dimensional gas chromatography (GC×GC-ToFMS) and (B) summary of the number of NTA features (Levels 1 and 2) per plastic sample.

NTA methods offer broader screening capability than target methods, although they are typically less sensitive. A summary of the NTA workflow used to analyze the samples and the number of features (Level 1 and 2) identified per plastic sample is presented in Figure 3. Over 5,000 features per sample were detected postprocessing using LC-HRMS and GC×GC/ToF-MS. Identification priority was placed on peaks that elicited mass spectral fragmentation with matches in spectral libraries/databases, implying many features outside these parameters would be overlooked. Identification and confirmation require further laborious structural elucidation efforts, including the application of *in silico* fragmentation tools. As a result, 191 peaks were tentatively identified as Level 2 compounds. Of these, 26 peaks were confirmed with authentic standards at the time of study (Level 1) (Figure 3B, Table S7). These included diethyltoluamide (DEET), a primary ingredient in insect repellant prevalent in surface waters,^37−39^ several phthalates, and PACs. Using the ClassyFire structural classification feature (http://classyfire.wishartlab.com/), benzenoids, lipids, and lipid-like molecules (e.g., straight-chain esters, phthalates, and PACs) were the dominant super classes (Figure S2-A). By class, fatty acyls, benzene, and substituted derivatives (e.g., benzoates) were the most dominant (Figure S2-B). These classes were previously reported to have higher incidences of occurrence in consumer products made from recycled materials compared to virgin products using suspect screening analysis.^12^

The wide range of analytes that were detected using NTA, additionally, included antioxidants and/or stabilizers, such as Irganox 1076 (Ix1076), Irgafos 168 (tris(2,4-di*tert*-butylphen-yl) phosphite (Ir168), oxidized Ir168 (Ox168), and 2,6-di-*tert*-butylphenol (DTBP) (DF > 80%) (Table S7). These are used in plastic production to minimize degradation from light or heat exposure.^40^ Benzophenone (BZP) and its derivatives, oxybenzone (OxB, benzophenone-3) and 4-methlybenzophenone (MBZP), were also detected in >95% of samples (Table S7). These chemicals have several industrial applications, including as UV-filters and photostabilizers in personal and plastic products.^41^ Furthermore, BZPs are noted for their environmental ubiquity,^42−44^ disruptive effects on coral reefs, and adverse health impacts in aquatic systems—which is a concern for all improperly disposed of plastic that directly enters aquatic ecosystems.^45,46^ Benzophenone (BZP) has been detected in both virgin and recycled polypropylene samples^47^ and recycled paper^48,49^ and was shown to have high migration potential into food items.^50^ Known as an endocrine disruptor, bisphenol A (BPA) was also detected in all samples and was similarly shown to be abundant in recycled HDPE pellets sampled from 24 countries worldwide.^51^

Due to the lack of authentic standards for many of the NTA chemicals, accurate quantification was not possible. Additionally, the method was not optimized for extraction, nor were extraction recoveries or matrix interferences considered. With these caveats and the focus of using a nonspecific extraction and screening method to study the samples, concentrations were estimated as outlined in the Materials and Methods. Estimated concentrations for individual chemicals ranged between 0 and 1,033 ng/g (Table S7). The highest estimated concentration was for 2-hexyl hydroxy benzoate (1,033 ng/g, 100% DF) classified as an odor agent. Other abundant and common chemicals included benzyl benzoate (840 ng/g, 95% DF), 2-benzyl hydroxy benzoate (716, ng/g 95% DF), BZP (514 ng/g, 100% DF), and 2 methoxy-naphthalene (or an isomer) (690 ng/g, 100% DF).

### Organic Chemicals and Current Regulations

The individual concentrations of organic chemicals went up to 3,850 ng/g (highest concentration for 2-ethylhexyl diphenyl phosphate, EHDPP). Several restrictions exist on the production and use of certain substances, such as PBDEs^52^ and DEHP,^53,54^ due to their persistence, bioaccumulation, and toxicity. This includes elimination directives by the Stockholm Convention on Persistent Organic Pollutants for some of these compounds,^55,56^ the 1000 mg/kg maximum allowable concentration by the European Directive on the Restriction of the Use of Certain Hazardous Substances in Electrical Equipment (RoHS),^57^ and the 1000 mg/kg low persistent organic pollutant concentration limit specified in Annex IV for BFR-contaminated waste.^58,59^ Additionally, the EU sets thresholds of no more than 500 mg/kg and 1000 mg/kg for unintentional trace contaminants (UTCs) in recycled plastics granules and flakes, respectively.^9,13,60,61^ Overall, the concentrations of organic chemicals detected in this study would be considered an “incidental presence”, meaning that sources appear to be a residual trace contaminant or impurity.

### Elements

**Figure 4:**
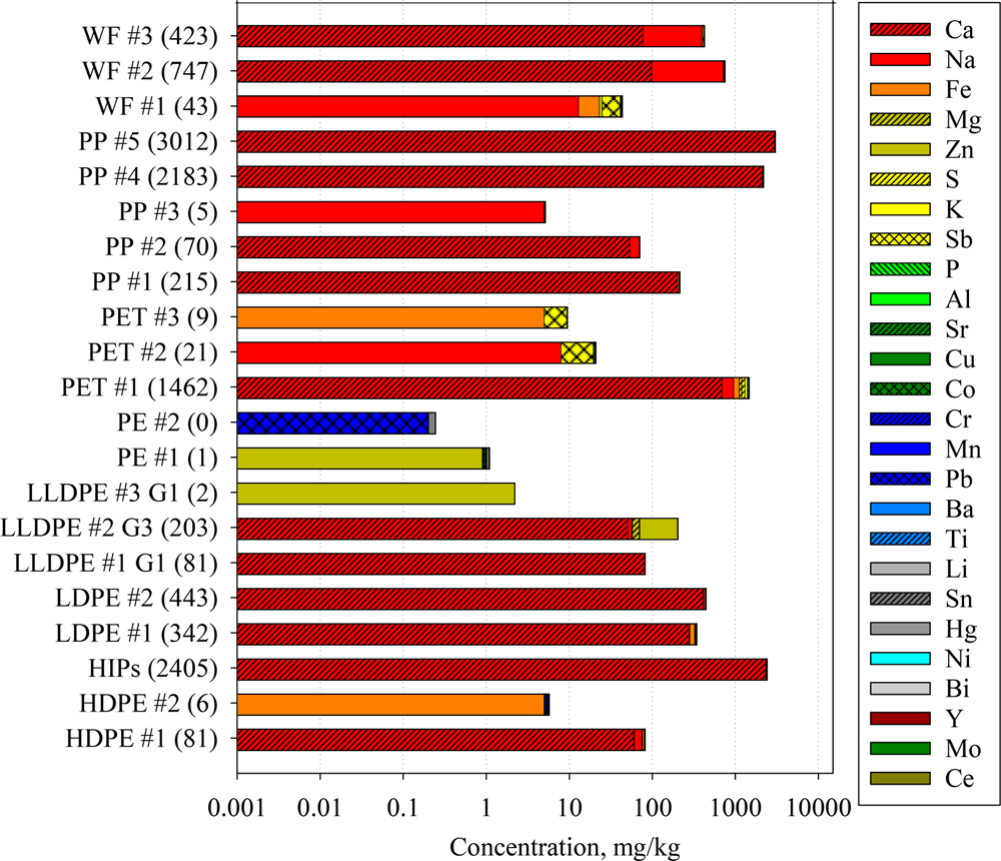
Concentrations (mg/kg) (stacked bar, *x*-axis) of elements detected per samples. Values in parentheses () represent total element concentrations in mg/kg in a given sample. Full names of plastic types and elements are provided in Tables 1 and S5, respectively.

Metal(loid)s have various applications in plastic production, including as metal-containing additives in stabilizers (e.g., Pb, Zn), catalysts (e.g., Sb, Hg, Cr), and coatings and pigments (e.g., Zn, Pb, Cr, Fe, and Co).^13,32,62−65^ In this study, 36 of the 60 elements analyzed were detected in at least one sample (Figures 4 and S9). The individual concentrations varied by almost 7 orders of magnitude (<0.005–2980 mg/kg), with the highest concentrations for calcium (Ca, 0–2980 mg/kg), sodium (Na, 0–617 mg/kg), and iron (Fe, 0–156 mg/kg) (Figure 4, Table S9). Mercury (Hg), zinc (Zn), lead (Pb), chromium (Cr), Ca, antimony (Sb), Na, strontium (Sr), Fe, and cobalt (Co) were the most frequently detected (∼50% DFs) (Figure 4, Table S9). In a prior study, titanium (Ti), copper (Cu), aluminum (Al), Fe, and Pb were the most frequently detected metals at concentrations exceeding 10 mg/kg (and up to 370 mg/kg) in washed and extruded LDPE recycled pellets.^27^

The concentrations of known neurotoxin Hg ranged from <0.01 to 0.487 mg/kg in the samples. In recycled paper products, Hg concentrations were reported within a similar range of 0.01 to 0.386 mg/kg.^66^ All samples were below the 0.3 mg/kg limit for Hg proposed for paper and board intended for use in food packaging applications,^67^ with the exception of one sample (PP#5 white pellets) (Table S9). Lead (Pb) concentrations were below the 3 mg/kg and 100 mg/kg legal limits for food contact materials and toys, respectively.^67,68^ Additionally, Pb and Cr concentrations were also below the 1000 mg/kg European legal limit value for electrical and electronic equipment.^57^

Although there was no apparent trend in analyte distribution according to resin type, PET samples had high concentrations of Sb (8.05 ± 3.55 mg/kg) (Figure 4, Table S9). PET samples were similarly shown to contain comparatively higher Sb levels (>100 mg/kg) compared to other polymers (i.e., PE, PP, PS) when various plastic materials, including household waste plastics, reprocessed plastic waste pellets and flakes, and virgin plastic were studied.^63^ The use of a catalyst containing antimony(III) trioxide (Sb_2_O_3_) during PET production,^69,70^ which is not expected to be present in the final product,^62^ was suggested as a potential source for the contamination.^63^

We detected seven rare earth elements (REEs) in at least one sample: cerium (Ce), europium (Eu), Gd (gadolinium), lanthanum (La), neodymium (Nd), praseodymium (Pr), and yttrium (Y) (Figure 4, Table S9). Generally, REE concentrations and DFs were low (Σ_7_REEs: <DL-0.406 mg/kg, 5–29% DFs). In a previous study, Σ_16_REEs concentrations of up to 8 mg/kg were reported in new and old consumer plastic products, including food contact materials, cosmetic containers, and office equipment.^71^ The presence of REEs in plastics is suspected to come from recycled electrical and electronic equipment plastics—as a result, they have been suggested as proxies to track unintentional contamination from e-waste.^69,71,72^

There are limited studies that profile or report metal(loids) in recycled plastics materials.^32,62,63,65^ However, results from a previous study suggested that reprocessed plastics could contain higher concentrations than virgin plastics.^63^ Additionally, several metals such as Zn, Ni, Fe, and Cu, are suspected as NIAS during the use and recycling of plastic.^13,27^ For these reasons, some studies petition that similar attention be paid to metal contamination in reprocessed or recycled plastics, as has been for organic chemicals and additives.^63,70,71^

#### Effect of Types (Flakes vs Pellets) and Color (Colored vs White/Natural)

**Figure 5:**
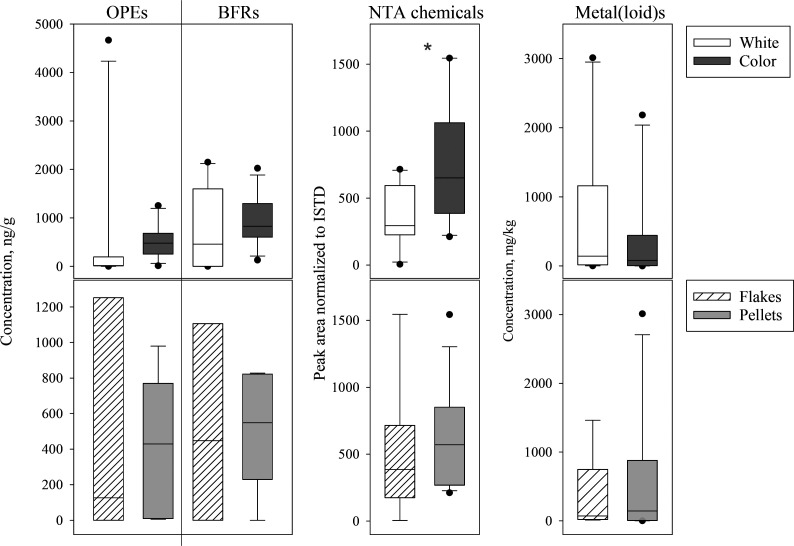
Concentrations and levels of all chemicals studied according to (top) color (white vs color) and (bottom) form (flakes vs pellets). Box plot extends from 25th to 75th percentile, with horizontal line representing the median. (*) represents the significant difference (*p* < 0.05).

Two-way ANOVA analysis with factors, form (pellets vs flakes), and color (colored vs white/naturals) showed that neither had an effect on the ΣOPEs, ΣBFRs, and Σelements concentrations (*p* > 0.4) (Figure 5). On the other hand, NTA chemicals showed variation with color (*p* = 0.03) (Figure 5). Another study that assessed color in recycled content reported higher levels of PACs in black LDPE pellets (vs white, brown, and green) and hypothesized the application of carbon black as a stabilizer as a probable source.^27,28^

Overall, the two-way ANOVA analysis results suggest no variation in chemical distribution between the flakes and the pellets. Ultimately, both will be used to manufacture recycled goods and products. Although hazardous substances have been reported in recycled goods,^3,12,20,21^ further studies are necessary to determine what proportion of the detected chemicals could be transferred to final products. Another factor we did not consider was the effect of resin type on chemical distribution due to limited representation of some polymers (e.g., HIPS, HDPE, etc.). Other factors such as the origin of plastic materials used to produce the flakes and pellets and processes used by different plastic companies and recyclers are likely to also contribute toward variation in chemical compositions and levels.

#### Limitations and Environmental Implications

In this study, we applied a broad screening approach of organic and inorganic chemicals in plastic flakes and recycled pellets intended for use in the manufacture of recycled products. This extraction method was as a result nonspecific and not optimized for certain chemical classes. The detection of many different types of substances suggests the extraction method approached our goal of being nondiscriminatory, allowing us to determine the presence of a large number of compounds in various recycled plastic materials. However, this approach presents a limitation as analyte detection and recovery may be impacted by chemical class and plastic type. Nevertheless, we collectively detected over 220 organic chemicals, including OPEs, phthalates, and benzophenones, and 36 elements in the plastic samples. These results provide pertinent information for the selection of chemicals in future targeted methods in recycled plastics. This includes spike and recovery experiments to address extraction and instrumental bias to determine accurate concentrations, using commercially available authentic standards (particularly for NTA tentatively identified compounds) and a more representative suite of labeled standards.

The individual concentrations of chemicals reported here would be considered residual or UTCs.^61,73^ This includes several substances that are associated with dose-dependent deleterious health effects, such as some OPEs which are known endocrine disruptors,^74^ and abundantly detected EHDPP and TNBP, which are linked with adverse developmental and reproductive outcomes in fish.^75,76^ Bisphenol A, which was detected in all samples, also has endocrine disrupting properties, with evidence for carcinogenic and mutagenic potential.^77^ Other known toxic chemicals detected include PACs and phthalates.^78,79^ Although we report many of these substances in the recycled plastic pellets and flakes, we cannot infer how many of these could end up in final products. In addition, we do not have a comparison to virgin plastic to examine the effect of recycling on contaminant load. Furthermore, these results cannot be extrapolated to determine an index of exposure which is necessary to evaluate risk due to harmful effects. We suggest further work, such as migration studies and effects-directed analysis approaches, to accurately characterize the toxicological burden from cumulative exposure to the many chemicals detected and identify chemicals of high concern to support risk assessment strategies.

As interest in the role of recycling in the circular economy model grows, other researchers recognize several challenges and barriers that could impede efforts to minimize the contamination of recycled products.^11^ These include the lack of transparency and traceability of substances of high concern between plastic manufacturers, the burden of financial costs on recyclers to decontaminate materials, the uncertainties in the regulations of NIAS or complex mixtures, the lack of refined and broad directives or legislation on recycled plastics in many countries, and the lack of guidelines on potentially hazardous substances in recycled plastics.^11^ The results of our study highlight that while recycling addresses sustainability goals, analysis of goods and products made from recycled products is required to fully understand the transfer of contaminants in a circular economy.

## Data Availability

All data needed to evaluate the conclusions in the paper are present in the paper and/or the Supporting Information. Additional data and information related to this paper are available upon request from the authors.

## Supporting Information Available

MS/MS Fragmentation patterns for selected antixodants matched to literature (Figure S1); superclass categories for chemicals detected using liquid chromatography Orbitrap mass spectrometry and comprehensive two-dimensional gas chromatography mass spectrometry, normalized to total area (Figure S2); list of halogenated flame retardants and perfluoroalkyl acids analyzed in recycled plastics in the study (Table S1); gas chromatography negative ion mass spectrometry conditions for the analysis of halogenated flame retardants (Table S2); GC×GC-ToFMS chromatogram oven temperature program (Table S3); data processing settings used in Compound Discoverer for peak detection and alignment (Table S4); elements analyzed in reclycled plastics using inductively coupled plasma–mass spectrometry and their detection limits (Table S5); list of chemicals used to estimate concentrations and/or confirm peaks in NTA (Table S6); chemical features detected in NTA using GCxGC/ToF-MS and LC-HRMS and their estimated concentrations (Table S7); detection frequencies and concentrations (minimum, maximum, mean, and standard deviations) of target organic chemicals in plastic recyclates (Table S8); and detection frequencies and concentrations (minimum, maximum, mean, and standard deviations) of elements in plastic recyclates (Table S9) (PDF)
