# Supplementary material for: Target and Nontarget Screening of Organic Chemicals and Metals in Recycled Plastic Materials
Source: Environ Sci Technol. 2023 Feb 14;57(8):3380–90. doi: 10.1021/acs.est.2c07254 (PMC9979653; doi:10.1021/acs.est.2c07254)
Supplement: Supplementary file 1 — es2c07254_si_001.pdf [file es2c07254_si_001.pdf]

SUPPORTING INFORMATION

Target and non-target screening of organic chemicals and metals in recycled plastic materials

Leah Chibwe,[1,2*#] Amila O. De Silva,[1] Christine Spencer,[1] Camilla F. Teixera,[1] Mary Williamson,[1] Xiaowa Wang,[1] Derek C.G. Muir*[1]

[1] Environment Climate Change Canada, Aquatic Contaminants Research Division, Burlington, ON L7S 1A1, Canada

[2] University of Regina, Institute for Environmental Change and Society, Regina, SK S4S0A2, Canada

*Corresponding authors: DCGM: Derek.Muir@ec.gc.ca and LC: leahchibwe@gmail.com

8 pages

2 Figures

9 Tables

Sample extraction. A rapid extraction method was implemented to process the plastic samples. The method was based on methodology used by the USEPA for screening neutral or semi-polar organic chemicals in consumer plastics.[1] Twenty mL of dichloromethane (DCM) was added to 5 g of plastic material (used as received) in a 40 mL-amber glass bottle; for the HIPs sample, 6% diethyl ether/hexane was used as the plastic melted in DCM. The internal standards phenanthrene-d10 (phe-d10) and chrysene-d12 (chr-d12) were added to the extracts for GC×GC-ToFMS analysis. With the exception of one sample (i.e., PP#5) which had unusual percent recoveries for chr-d12 (335 %), phe-d10 (285 %), percent recoveries were between 68-174 % (107±29 %) and 62-177 chr-d12 and phe-d10, respectively. Samples were then further sonicated for 1 hr, then left on a mixer overnight. The following day, samples were sonicated for 1 hr, vortexed and sonicated for another 15 min. The remaining solvent was transferred to clean vials and proportioned into 4-5 mL aliquots. Prior to LC-HRMS analysis, labelled internal standards (ISTDs) perfluoro-n-[1,2-13C2]tetradecanoic acid (M2PFTeDA) and sodium perfluoro-1-(13C8)octanesulfonate (M8PFOS), and tris(2-chloroisopropyl)phosphate-d18 (d18TCPP) were added to the extracts in negative and positive mode, respectively. Recoveries for these ISTDs were between 85-106 % (99±5 %) (M2PFTeDA) and 89-104 % (100±3%), (M8PFOS), and 89-104 % (99±3 %), (d18TCPP), although it should be noted that these values do not reflect loss during extraction and clean-up.

Extraction of perfluoroalkyl acids (PFAAs) was carried out separately as these ionizable substances were not expected to be extracted with DCM or diethylether/hexane. The methodology followed Lescord et al (2015) with minor modifications.[2] The plastic materials (5g; used as received) were shaken (30 min) with acetonitrile (ACN) and centrifuged. A suite of isotopically labeled (^{13}C) internal standards of PFAAs (C4-C14 perfluorocarboxylates (PFCAs);

C4-C12 perfluoroalkyl sulfonates (PFSAs); Wellington Laboratories, Guelph ON) were added at the extraction step. The ACN was evaporated to dryness under N_2 and reconstituted in 1.0 mL of methanol (MeOH). Samples were passed through pre-conditioned (5.0 mLMeOH and 2.0 mL glacial acetic acid) carbon SPE columns (Supelclean ENVI-Carb 1 mL 100 mg) and eluted using MeOH. The resulting extracts were dried and reconstituted to 1.0 mL in a 50:50 solution of methanol:water for LC-MS/MS analysis. Recoveries of spiked native C4-C14 PFCAs and C4-C12 PFSAs averaged 87% (range 75 to 127%).

Non-target Screening and Identification. For GC×GC-ToFMS, analysis was conducted in electron ionization mode using a GC × GC/ToF-MS Pegasus 4D (LECO, St Joseph, MI) equipped with an Agilent 7890B gas chromatograph (Palo Alto, CA). The columns used were the DB-1 MS UI (25 m × 0.25 mm × 0.25 µm; Sigma-Aldrich) and Rxi-17 Sil MS column (1.2 m × 0.25 mm × 0.25 µm; Restek) in the first and second dimension, respectively. Further information on oven program methods and other operational parameters are provided in the Supporting Information **(Table S3).** Data was processed using the LECO ChromaToF v.4.50.8 software and included peak deconvolution and background subtraction. Recoveries for phenanthrene-d10 and chrysene-d12, spiked in plastics prior to extraction were between 71-95% across the various plastic types. Tentative identification was conducted using the NIST 11 EI mass spectral library (library score >85%). Peaks were critically evaluated for isotope patterns and mass library matches. Analyte peaks with poor chromatography in any given sample were excluded from further analysis

For LC-HRMS, chemical analysis was performed on a Thermo Vanquish ultra-high-performance liquid chromatograph coupled to a Q Exactive Focus Orbitrap MS (Thermo Fisher Scientific, Mississauga, ON) using an Acquity UPLC BEH C_{18} column (2.1 mm × 50 mm, 1.7

μm particle size (Waters, Milford, MA, U.S.A.) for chromatographic separation. The mobile phases were HPLC grade water (A) and methanol (B). The injection volume and flow rate were 5 μL, and 300 μL/min, respectively. Full scan data-dependent acquisition discovery mode (Full MS/dd-ms^2) was used at a resolution of 70,000 FWHM (Full) and 35,000 FWHM (ms^2) and a mass range of 100–1700 *m*/z in electrospray ionization mode (ESI) positive and negative mode. LC-HRMS data were processed using the Compound Discoverer software and processing parameters are provided in the Supporting Information **(Table S4).** Peaks were tentatively identified using exact masses (Δ mass <5 ppm), isotopic profile fit > 70%, and MS^2 fragmentation experimental match with and mzCloud mass spectra library or literature. We considered this as a reasonably high degree of confidence identification in lieu of authentic standards.[3]

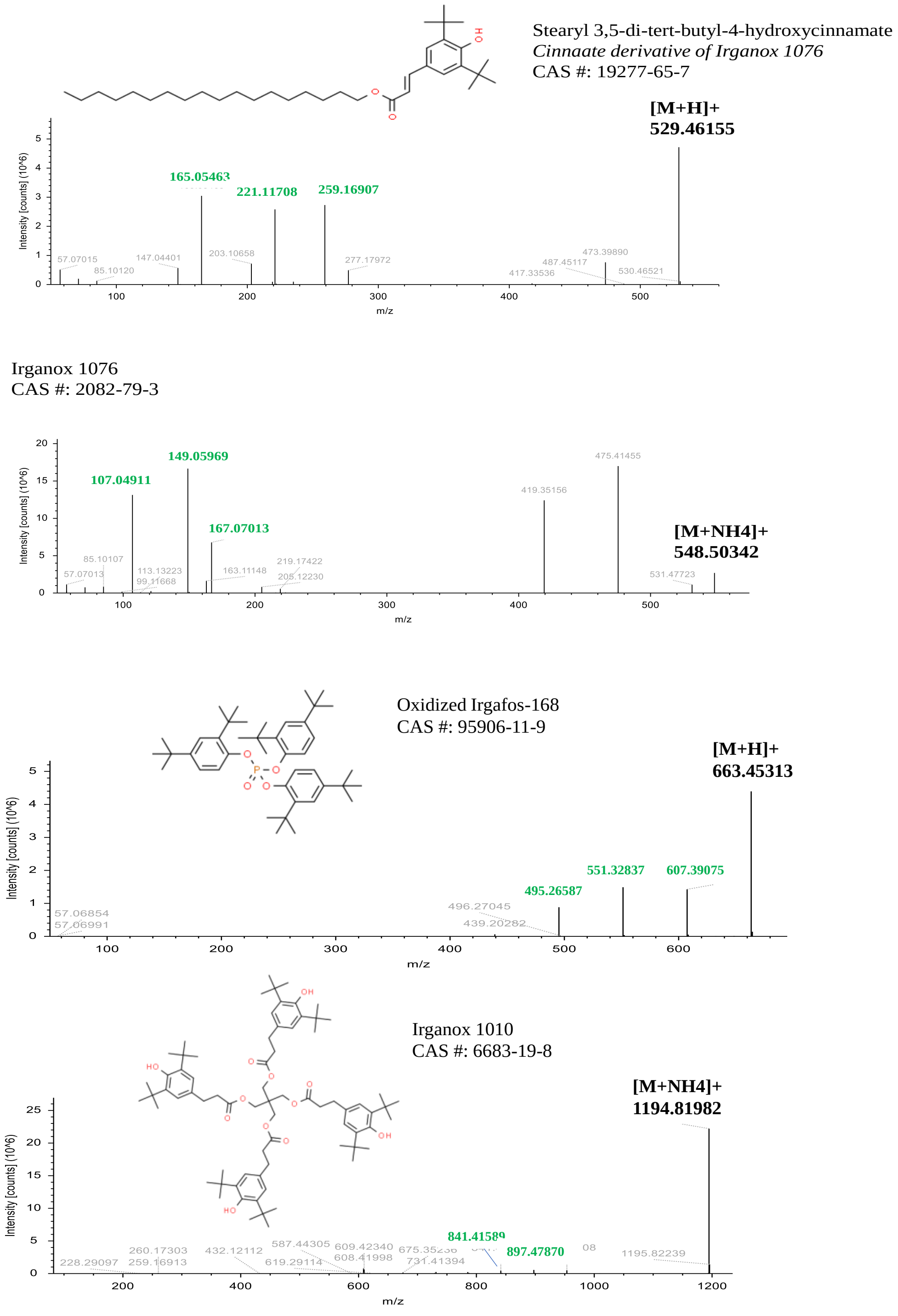

Figure S1. MS/MS Fragmentation match to literature. Fragments in **bold green** represent matched fragments to literature.[4]

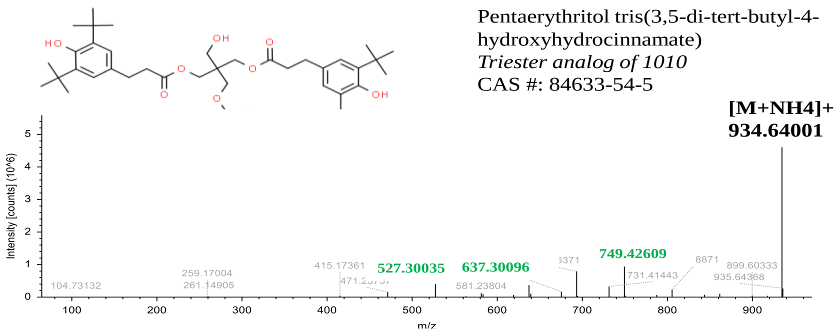

Figure S1 cont. MS/MS Fragmentation match to literature. Fragments in **bold green** represent matched fragments to literature.[4]

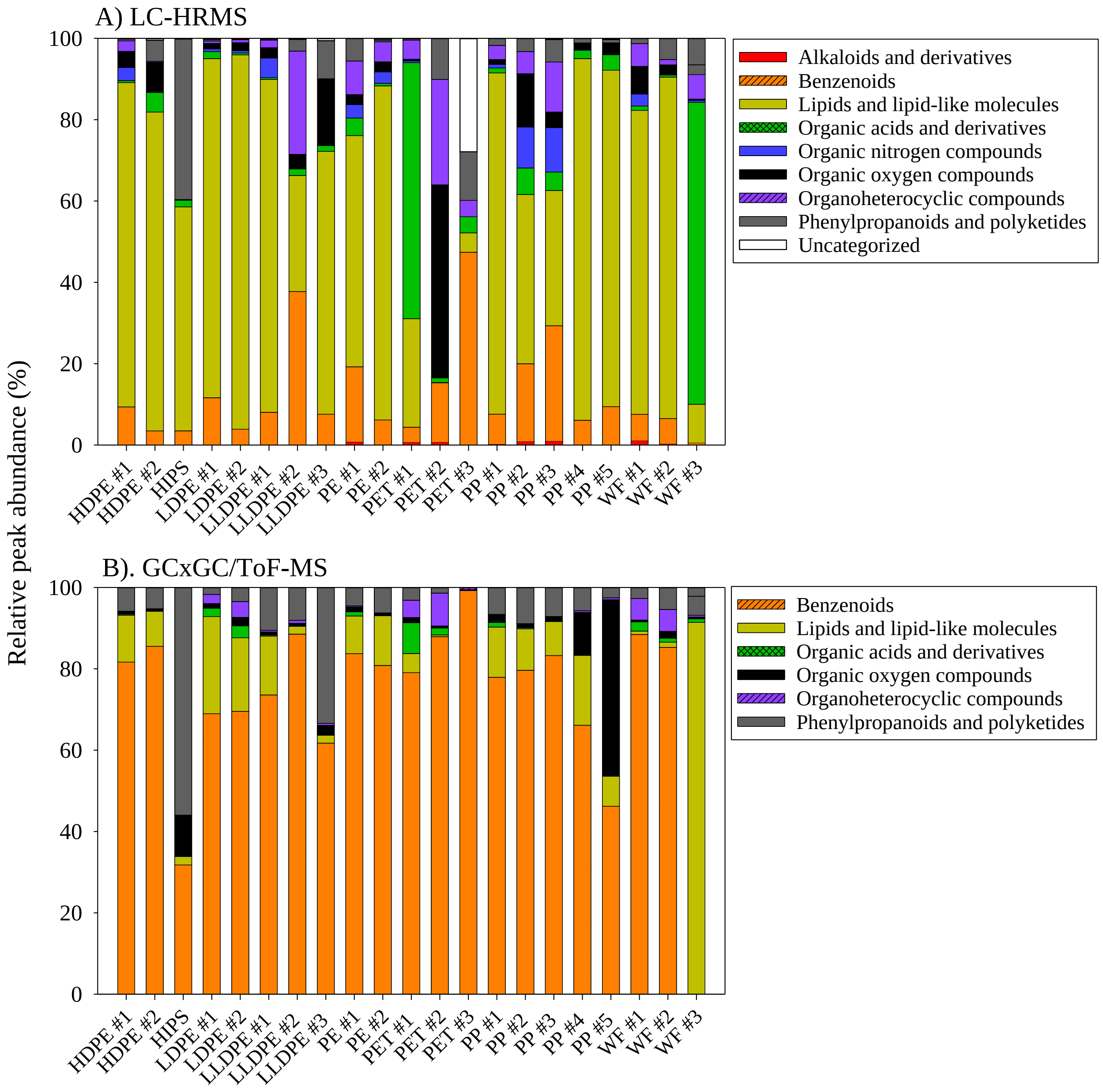

Figure S2-A. Superclass categories for chemicals detected using (A) liquid chromatography Orbitrap mass spectrometry and (B) comprehensive two-dimensional gas chromatography mass spectrometry, normalized to total area. Class categories were determined using the ClassyFire web application (http://classyfire.wishartlab.com/

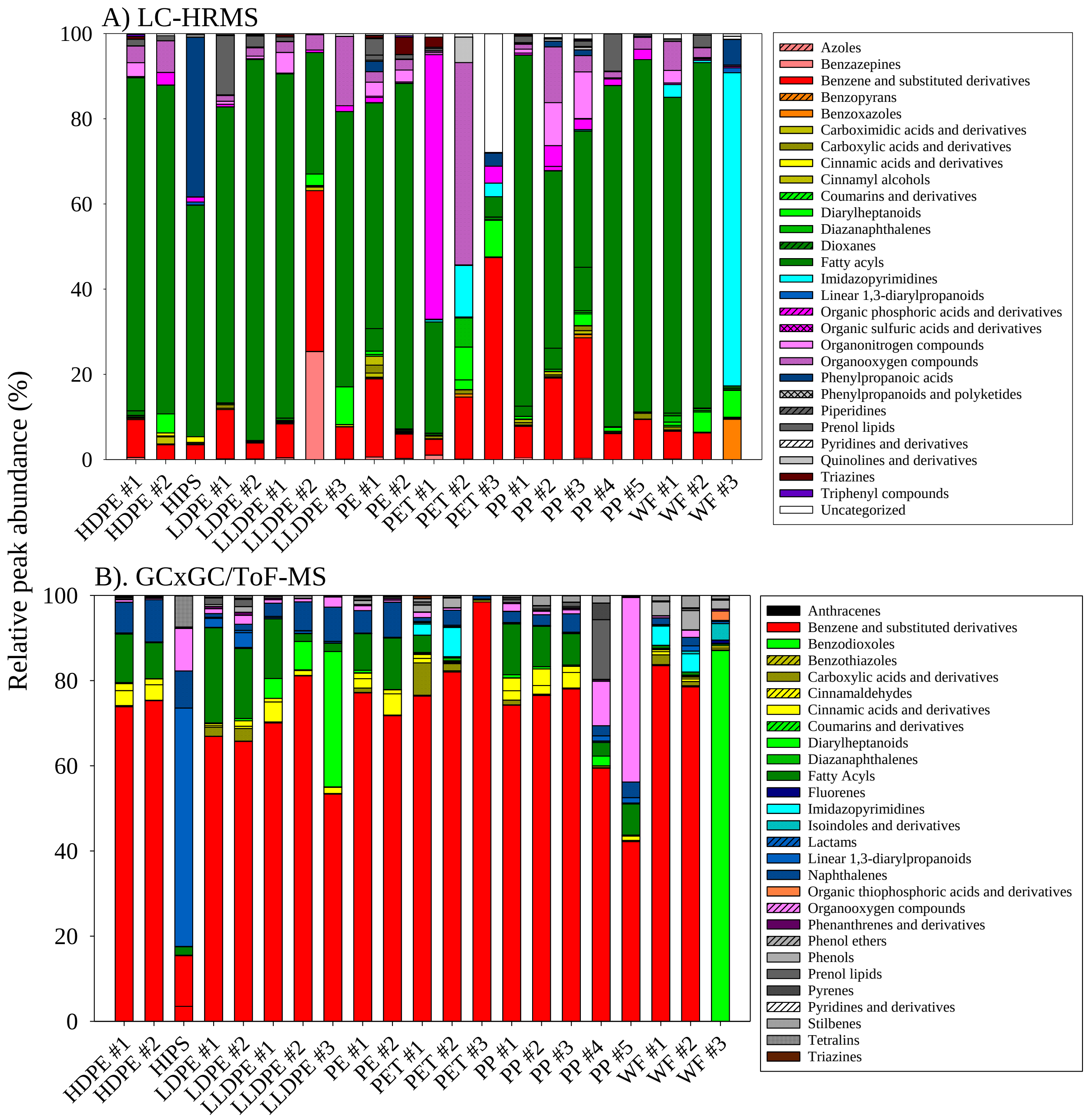

Figure S2-B. Class categories for chemicals detected using (A) liquid chromatography Orbitrap mass spectrometry and (B) comprehensive two-dimensional gas chromatography mass spectrometry. Class categories were determined using the ClassyFire web application (http://classyfire.wishartlab.com/)

Table S1. Halogenated flame-retardants and perfluoroalkyl acids analyzed in recycled plastics in the study.

Name	Chemical name	Chemical Abstracts Registry #	Ions monitored (m/z)
	Polybrominated diphenyl ethers (PBDEs)		
BDE-10	2,6-Di	51930-04-2	
BDE-7	2,4-Di	171977-44-9	
BDE-11+8	3,3'-Di	6903-63-5	
BDE-12+13	3,4-/3,4'-Di	189084-59-1, 83694-71-7	
BDE-15	4,4'-Di	2050-47-7	
BDE-30	2,4,6-Tri	155999-95-4	
BDE-32	2,4',6-Tri	155999-95-4	
BDE-17+25	2,2',4-/2,3',4-Tri	147217-75-2, 337513-67-4	79, 81, 327
BDE-28+33	2,4,4'-/2',3,4-Tri	41318-75-6, 147217-78-5	79, 81, 327
BDE-35	3,3',4-Tri	147217-80-9	
BDE-37	3,4,4'-Tri	147217-81-0	
BDE-75	2,4,4',6-Tetra	189084-63-7	
BDE-49	2,2',4,5'-Tetra	243982-82-3	79, 81, 325
BDE-71	2,3',4',6-Tetra	189084-62-6	79, 81, 325
BDE-47	2,2',4,4'-Tetra	5436-43-1	79, 81, 325
BDE-66	2,3',4,4'-Tetra	189084-61-5	79, 81, 325
BDE-77	3,3',4,4'-Tetra	93703-48-1	
BDE-100	2,2',4,4',6-Penta	189084-64-8	79, 81, 403
BDE-119	2,3',4,4',6-Penta	189084-66-0	
BDE-99	2,2',4,4',5-Penta	60348-60-9	79, 81, 403
BDE-118	2,3',4,4',5-Penta	446254-80-4	
BDE-116	2,3,4,5,6-Penta	189084-65-9	
BDE-85	2,2',3,4,4'-Penta	182346-21-0	79, 81, 403
BDE-126+155	3,3',4,4',5-Penta/2,2',4,4',6,6'-Hexa-	366791-32-4/35854-94-5	
BDE-154	2,2',4,4',5,6'-Hexa	207122-15-4	79, 81, 403
BDE-153	2,2',4,4',5,5'-Hexa	68631-49-2	79, 81, 403
BDE-138+166	2,2',3,4,4',5'-/2,3,4,4',5,6-Hexa	182677-30-1, 189084-58-0	79, 81, 484/403
BDE-183	2,2',3,4,4',5',6-Hepta	207122-16-5	79, 81, 484
BDE-181	2,2',3,4,4',5,6-Hepta	189084-67-1	
BDE-190	2,3,3',4,4',5',6-Hepta	446255-30-7	79, 81
BDE-202	2,2',3,3',5,5',6,6'-Octa	67797-09-5	79, 81
BDE-201	2,2',3,3',4,5',6,6'-Octa	32536-52-0	79, 81

BDE-204+197	2,2',3,4,4',5,6,6'-/2,2',3,3',4,4',6,6'-Octa	446255-54-5, 117964-21-3	79, 81
BDE-198+199+200+203	2,2',3,3',4,5,5',6'-/ 2,2',3,3',4,5,5',6-/ 2,2',3,3',4,5,6,6-/ 2,2',3,4,4',5,5',6-octa	32536-52-0, 446255-50-1, 337513-72-1	79, 81
BDE-196	2,2',3,3',4,4',5,6'-Octa	446255-39-6	79, 81
BDE-203	2,2',3,4,4',5,5',6-Octa	337513-72-1	79, 81
BDE-194	2,2',3,3',4,4',5,5'-Octa	32536-52-0	79, 81
BDE-195	2,2',3,3',4,4',5,6-Octa	446255-38-5	79, 81
BDE-208	2,2',3,3',4,5,5',6,6'-Nona	437701-78-5	
BDE-207	2,2',3,3',4,4',5,6,6'-Nona	437701-79-6	
BDE-206	2,2',3,3',4,4',5,5',6-Nona	63936-56-1	
	Brominated flame retardants (BFRs)		
ATE	Allyl 2,4,6-tribromophenyl ether	3278-89-5	290.8, 79, 81
BATE	2-Bromoallyl 2,4,6-tribromophenyl ether	99717-56-3	79, 81, 159.8
P-TBX	2,3,5,6-Tetrabromo-p-xylene	23488-38-2	79, 81
PBBZ	Pentabromobenzene	608-90-2	
TBCT	tetrabromo-o-chlorotoluene	39569-21-6	
PBT	Pentabromotoluene	87-83-2	487.6, 485.6, 81
PBEB	Pentabromoethylbenzene	85-22-3	79, 81, 499.6
DPTE	2,3-Dibromopropyl 2,4,6-tribromophenyl ether	35109-60-5	290.8, 81, 159.8
HBB	Hexabromobenzene	87-82-1	551.5, 471.6, 79
BB-101	2,2',4,5,5'-Pentabromobiphenyl	67888-96-4	
PBBA	Pentabromophenyl-methyl acrylate	59447-55-1	
EHTBB	2-ethyl-1-hexyl 2,3,4,5-tetrabromobenzoate	183658-27-7	468.8, 356.6, 81
g-HBCD	Hexabromocyclododecane	3194-55-6	79, 81, 159.9, 542.8
BTBPE	1,2-Bis(2,4,6-tribromophenoxy)ethane	37853-59-1	79. 81, 250.7
BEHTBP	Bis(2-ethyl-1-hexyl)tetrabromophthalate	26040-51-7	512.7, 383.7, 79
O-BIND	Octabromotrimethylphenyl indane	155613-93-7	
S-DP	Syn-Dechlorane Plus	135821-03-3	583.7, 509.8, 236.8
A-DP	Anti-Dechlorane Plus	13560-89-9	583.8, 547.7, 236.7
	Perfluoroalkyl acids (PFAS)		
PFBA	Perfluorobutanoic acid	375-22-4	213 > 169
PFPeA	Perfluoropentanoic acid	2706-90-3	263 > 219
PFHxA	Perfluorohexanoic acid	307-24-4	313 > 269
PFHpA	Perfluoroheptanoic acid	375-85-9	363 > 319
PFOA	Perfluorooctanoic acid	335-67-1	413 > 369
PFNA	Perfluorononanoic acid	375-95-1	463 > 419
PFDA	Perfluorodecanoic acid	335-76-2	513 > 469
PFUnA	Perfluoroundecanoic acid	2058-94-8	563 > 519
PFDoA	Perfluorododecanoic acid	307-55-1	613 > 569
PFTriA	Perfluorotridecanoic acid	72629-94-8	663 > 619
PFTeDA	Perfluorotetradecanoic acid	376-06-7	713 > 669
PFHxDA	Perfluorohexadecanoic acid	67905-19-5	813 > 769
PFBS	Perfluorobutanesulfonoic acid	375-73-5	299 > 99
PFHxS	Perfluorohexanesulfonoic acid	355-46-4	399 > 99
PFHpS	Perfluoroheptanesulfonoic acid	375-92-8	449 > 99

PFOS	Perfluorooctanesulfonoic acid	1763-23-1	499 > 99
PFOS-L	Linear-Perfluorooctanesulfonoic acid		515 > 99
PFDS	Perfluorodecanesulfonoic acid	335-77-3	599 > 99
PFOSA	Perfluorooctanesufonamide	754-91-6	498 > 78
PFECHSb	Perfluoroethylcyclohexane sulfonate	335-24-0	461 > 381
CL PFOS	Chloroperfluorooctanesulfonic acid	NA	
	Organophosphorus flame retardants (OPFRs)		
TMP	Trimethyl phosphate	512-56-1	141 > 109
TEP	Triethyl phosphate	78-40-0	183 > 99
TBPO	Tributylphosphine oxide	814-29-9	219 >92
TPrP	Tri-propyl phosphate	513-08-6	225 > 99
TNBP	Tributyl phosphate	126-71-6	267 > 99
TIBP	Triisobutyl phosphate	126-71-6	267 > 99
TPPO	Triphenylphosphine oxide	791-28-6	279 > 77
TCEP	Tris(2-chloroethyl)phosphate	115-96-8	285 > 99
TEEDP	Tetraethylethylene diphosphonate	995-32-4	303 > 219
TCPP	Tris(2-chloroisopropyl) phosphate	13674-84-5	327 > 99
TPHP	Triphenyl phosphate	115-86-8	327 > 77
EHDPP	2-ethylhexyl diphenyl phosphate	1241-94-7	363 > 77
o-TOTP	Tri-o-totyl phosphate (tricresyl phosphate isomers)	1330-78-5	369 > 166; 369 >91
m,p-TOTP	Tri-m and p-totyl phosphate (tricresyl phosphate isomers)		
DIPIPP	Diphenyl-3-isopropylphenyl phosphate	69515-46-4	369 > 233; 369 > 327
DOPP	Dioctylphenyl phosphonate	1754-47-8	383 > 141; 383 > 159
TBDPP	(4-tert-butylphenyl) diphenyl phosphate	56803-37-3	383 > 152; 383 > 215
IDDPP	Isodecyl diphenyl Phosphate	29761-21-5	391 > 251
TBOEP	Tris(2-butoxyethyl) phosphate	78-51-3	399 > 199; 399 > 299
TDCIPP	Tris(1,3-dichloroisopropyl)phosphate	13674-87-8	429 > 99; 431 > 99
TEHP	Tris(2-ethylhexyl)phosphate	78-42-2	435 > 71; 435 > 99
DTBPPP	Bis(p-tert-butylphenyl) phenyl phosphate	115-87-7	439 > 327; 439 > 383
T2IPPP	Tris(2-isopropylphenyl)phosphonate	64532-95-2	453 > 327; 453 > 369
TTBPP	tris(p-tert-Butylphenyl) phosphate	78-33-1	495 > 327; 495 > 439
TDBPP	Tris(2,3-dibromopropyl) phosphate	126-72-7	697 > 99; 699 > 99

Table S2. Gas chromatography negative ion mass spectrometry conditions for the analysis of halogenated flame-retardants.

**INSTRUMENT AND EQUIPMENT**	
Instrument	GC-MSD (6890 GC - 5973 MSD) (Agilent Technologies, U.S.A.)
MSD Operation mode	Selected Ion Mode (SIM)
Ionisation Method	Negative Chemical Ionisation (NCI)
Capillary Column	HP-5MS (J&W, (5%-Phenyl)-methylpolysiloxane, 30 m, 0.25 mm i.d., 0.1 µm film thickness)
	RTX-1614 (Restek, 5% diphenyl, 95% dimethyl polysiloxane, 30 m, 0.25 mm i.d., 0.1 µm film thickness)
Carrier gas	Helium at 1 mL/min
Sample Injection	Pulsed-splitless mode, 276 kPa for 1 min
Solvent delay	5 min.
Sample Injection Volume	2 µL
**TEMPERATURES**	
Injector	220 ºC
Ion Source	250 ºC
Quadrupole	150 ºC
Transfer Line	330 ºC
Oven	80ºC, hold 2 min, 7ºC min^{-1} to 110ºC, 3ºC min^{-1} to 250ºC, 10ºC min^{-1} to 285ºC, hold 12 min

Table S3. GC×GC-ToFMS chromatogram oven temperature program.

	**DB1 × Rxi17**
Injection volume	1 µL
1D column	DB-1 MS UI (25 m × 0.25 mm × 0.25 µm)
2D column	Rxi-17 Sil MS column (1.2 m × 0.25 mm × 0.25 µm)
Total run time	37 min
Carrier gas	He
Inlet mode	Splitless
Flow	1 mL min-1
Inlet Temp.	300 °C
1D Oven	60 °C (2 min), 15 °C min-1 to 150 °C, 6 °C min-1 to 260 °C (2 min), 15 °C min-1 to 310 °C (5 min)
2D Oven	85 °C (2 min), 15 °C min-1 to 175 °C, 6 °C min-1 to 285°C (2 min), 15 °C min-1 to 335 °C (5 min)
Modulator Temp. Offset	15 °C
Modulation period	5 s
Hot pulse	1.2 s
Cool time	1.3 s
Transfer line Temp	260 °C
Mass range	40-500 u
Acquisition rate	200 spectra s-1
Ion Source Temp	250 °C
Chiller Temp	- 80 °C

Table S4: Data processing settings used in Compound Discoverer for peak detection and alignment.

	ESI +ve	ESI -ve
Select Spectra		
Lower RT Limit	0.5	0.5
Upper RT Limit	20	20
Min Precursor Mass, Da	100	100
Max Precursor Mass, Da	2000	2000
Total Intensity Threshold		5000
Align RT times		
Alignment model	Adaptive	Adaptive
Mass Tolerance, ppm	5	5
Max. shift [min]	0.5	0.5
Detect Compounds		
General Settings		
Mass Tolerance, ppm	5	5
Intensity Tolerance, %	30	30
S/N Tolerance	6	6
Min Peak Intensity	100000	100000
Ions	[M+H]+1	[M-H]-1
Min Element Counts	CH	CH
Max Element Counts	C80 H120 Br15 CL15 F30 N10 O15 P5 S10	C80 H120 Br15 CL15 F30 N10 O15 P5 S11
Group compounds		
Compound Consolidation		
Mass Tolerance, ppm	5	
RT Tolerance [min]	0.1	
Best compound selection		
Rule #1	MS Order	
Rule #2	Ion type	
Preferred MS order	MS1	
Preferred ion	[M+H]+1	

	ESI +ve	ESI -ve
Fill Gaps		
Mass Tolerance, ppm	5	5
S/N Threshold	5	5
Mark Background Compounds		
Max Sample/Blank	5	5
Predict Composition		
Prediction settings		
Mass Tolerance, ppm	5	6
Min Element Counts	CH	CH
Max Element Counts	C80 H120 Br15 CL15 F30 N10 O15 P5 S10	C80 H120 Br15 CL15 F30 N10 O15 P5 S11
Min RDBE	-40	-40
Max RDBE	40	40
Min H/C	0.1	0.1
Max H/C	3	3
Max # candidates	10	10
Pattern Matching		
Intensity Tolerance, %	30	30
Intensity Threshhold, %	0.1	0.1
S/N Threshold	*3*	*3*
Min spectral fit, %	10	
ddMS2 mzCloud search		
Compound Classes	All	All
Match ion activation type	FALSE	FALSE
Match ion activation energy	Match with tolerance	Match with tolerance
Apply intensity threshold	TRUE	TRUE
Identity search	HighChem High Res	HighChem High Res
Similarity search	Confidence forward	Confidence forward
Match factor threshold	50	51

Table S5. Metal(loid)s analyzed in recycled plastics using inductively coupled plasma-mass spectrometry and their detection limits.

Metal(loid)s	Abbrv.	RDL	Metal(loid)s	Abbrv.	RDL
Aluminum	Al	20	Neodymium	Nd	0.005
Antimony	Sb	0.01	Nickel	Ni	0.2
Arsenic	As	0.02	Niobium	Nb	0.05
Barium	Ba	0.5	Palladium	Pd	0.02
Beryllium	Be	0.005	Phosphorus	P	20
Bismuth	Bi	0.005	Platinum	Pt	0.01
Boron	B	5	Potassium	K	20
Cadmium	Cd	0.1	Praseodymium	Pr	0.005
Calcium	Ca	50	Rhodium	Rh	0.002
Cerium	Ce	0.02	Rubidium	Rb	0.05
Cesium	Cs	0.005	Ruthenium	Ru	0.01
Chromium	Cr	0.05	Samarium	Sm	0.005
Cobalt	Co	0.01	Scandium	Sc	0.02
Copper	Cu	0.2	Selenium	Se	0.02
Europium	Eu	0.005	Silver	Ag	0.005
Gadolinium	Gd	0.005	Sodium	Na	5
Gallium	Ga	0.01	Strontium	Sr	0.05
Germanium	Ge	0.02	Sulfur	S	20
Hafnium	Hf	0.05	Tellurium	Te	0.02
Holmium	Ho	0.005	Terbium	Tb	0.005
Iridium	Ir	0.01	Thallium	Tl	0.005
Iron	Fe	5	Tin	Sn	0.1
Lanthanum	La	0.01	Titanium	Ti	1
Lead	Pb	0.02	Tungsten	W	0.02
Lithium	Li	0.05	Uranium	U	0.002
Lutetium	Lu	0.005	Vanadium	V	0.1
Magnesium	Mg	10	Ytterbium	Yb	0.005
Manganese	Mn	0.5	Yttrium	Y	0.01
Mercury, Extractable	Hg	0.01	Zinc	Zn	0.5
Molybdenum	Mo	0.02	Zirconium	Zr	0.2

Table S6. List of chemicals used to estimate concentrations and/or confirm peaks in non-targeted analysis.

Chemical	Exact mass	CAS #	Log Kow	Range (pg/ul)	R^2
GC×GC-ToFMS					
Triethylphosphate	182.07080	78-40-0	0.8	5-700	0.999
Dimethyl phthalate	194.05791	131-11-3	1.6	0.1-700	0.999
Tripropyl phosphate	224.11775	513-08-6	1.87	0.1-700	0.999
Benzothiazole	135.01427	95-16-9	2.01	0.1-700	0.997
Indole	117.05785	120-72-9	2.14	0.1-700	0.999
Diethyltoluamide	191.13101	134-62-3	2.18	0.25-700	1.000
2-aminonapthalene	143.07350	91-59-8	2.28	5-700	1.000
Diethyl phthalate	222.08921	84-66-2	2.47	0.1-700	0.999
6-Methyl-Quinoline	143.07350	91-62-3	2.57	0.25-700	0.999
p-Ethylphenol	122.07316	123-07-9	2.58	0.1-700	0.995
Phenazine	180.06875	92-82-0	2.84	0.1-700	0.999
4-Isopropylphenol	136.08882	99-89-8	2.9	0.1-700	0.996
2,6-Dimethylquinoline	157.08915	877-43-0	3.24	0.1-700	1.000
Naphthalene	128.06260	91-20-3	3.3	0.1-700	0.997
4-tert-Butylphenol	150.10447	98-54-4	3.31	0.1-700	0.998
Acridine	179.07350	260-94-6	3.4	0.5-700	0.994
Phenanthridine	179.07350	229-87-8	3.48	0.5-700	1.000
4H-Benzo[def]carbazole	191.07350	203-65-6	**3.82**	0.5-700	0.995
Acenaphthene	154.07825	83-32-9	3.92	0.1-700	0.997
Acenaphtheylene	152.06260	208-96-8	3.94	0.1-700	0.998
Benzyl Benzoate	212.08373	120-51-4	3.97	0.25-700	1.000
Tributyl phosphate	266.16470	126-73-8	4	0.1-700	0.999
Fluorene	166.07825	86-73-7	4.18	0.25-700	0.999
2,6-Dimethylnaphthalene	156.09390	581-42-0	4.31	0.1-700	0.998
1,5-Dimethyl naphthalene	156.09390	571-61-9	4.38	0.1-700	1.000
Dibenzothiopene	184.03467	132-65-0	4.38	0.25-700	1.000
11H-Benzo[a]carbazole	217.08915	239-01-0	**4.41**	0.1-700	1.000
Phenanthrene	178.07825	1985-01-08	4.46	0.25-700	0.999
Benz[a]acridine	229.08915	225-11-6	4.48	0.5-700	0.991
Benz[c]acridine	229.08915	225-51-4	4.49	0.5-700	0.994
Dibutyl phthalate	278.15181	84-74-2	4.5	0.1-700	0.999
Benzyl butyl phthalate	312.13616	85-68-7	4.73	5-700	0.996
Octa-fluoro-naphthalene	271.98723	313-72-4	**4.77**	0.5-700	1.000
Phenol, 2,6-bis(1,1-dimethylethyl)-	206.16707	128-39-2	4.92	0.25-700	0.999

1-Methyl fluorene	180.09390	1730-37-6	4.97	0.25-700	1.000
2-Methyl anthracene	192.09390	613-12-7	5	0.5-700	0.998
9-Methyl anthrancene	192.09390	779-02-2	5.07	1-700	0.996
1-Methylphenanthrene	192.09390	832-69-9	5.08	0.5-700	0.996
2,6-Di-tert-butyl-4-methylphenol	220.18272	128-37-0	5.1	0.5-700	0.999
2,4-Di-tert-butylphenol	206.16707	96-76-4	5.19	0.1-700	0.999
Benzo[b]fluorene	216.09390	14458-76-5	5.28	5-700	0.997
3,6-Dimethyl phenantherene	206.10955	1576-67-6	5.44	0.25-700	0.998
Dicyclohexyl phthalate in Hex	330.18311	84-61-7	5.6	0.5-700	0.995
Dibenz[a,j]acridine	279.10480	224-42-0	5.63	5-700	0.997
Benzo[b]fluoranthene	252.09390	205-99-2	5.78	5-700	0.996
Chrysene	228.09390	218-01-9	5.81	0.5-700	0.995
2,4,6-Tri-tert-butylphenol	262.22967	732-26-3	6.06	0.5-700	1.000
Benzo[a]pyrene	252.09390	50-32-8	6.13	5-700	0.997
Retene	234.14085	483-65-8	**6.35**	5-700	0.997
Benzo[ghi]perylene	276.09390	191-24-2	6.63	5-700	0.997
Dibenzo[a]anthracene	278.10955	53-70-3	6.75	5-700	0.996
Octabenzone	326.18819	1843-05-6	6.96	1-700	0.992
Dioctyl phenylphosphonate	382.26368	1754-47-8	**7.51**	0.5-700	0.998
Di-n-octyl phthalate	390.27701	117-84-0	8.1	0.1-700	0.997
3,6-Dimethyl-9H-carbazole	195.10480	5599-50-8	na	0.25-700	0.995
2,4-Dimethylbenzo[h]quinoline	207.10480	605-67-4	na	10-700	0.999
LC-HRMS POS					
5-Methyl-1H-benzotriazole	134.0718272	136-85-6	1.71	0.1-800	0.996
Triethylphosphate	183.078626	78-40-0	0.8	0.1-800	0.998
Dimethyl phthalate	195.0657388	131-11-3	1.6	0.1-800	1.000
2,4,4'-Trihydroxybenzophenone	231.0657388	1470-79-7	2.48	0.1-800	0.999
Benzothiazole	136.0221003	95-16-9	2.01	0.1-800	0.999
5,6-Dimethyl-1H-benzotriazole	148.0874773	4184-79-6	2.26	0.1-800	0.999
Tris(2-chloroethyl) orthophosphate	284.961709	115-96-8	1.44	0.1-800	0.999
Tris(2-chloroethyl)phosphite	268.966794	140-08-9	1.51	5-800	0.997
Diethyltoluamide	192.1388442	134-62-3	2.26	0.1-800	0.990
2,4-Dihydroxybenzophenone	215.0708242	131-56-6	2.96	0.1-800	0.999
Phenazine	181.0765783	92-82-0	2.84	0.1-800	1.000
Dioxybenzone	245.0813889	131-53-3	3.82	0.1-800	0.998
Carbazole	168.0813293	86-74-8	3.72	0.5-800	0.999
9-Fluorenone	181.0653449	486-25-9	3.58	0.1-800	0.999
Benzothiazole, 2-(methylthio)-	182.0098216	615-22-5	3.15	5-800	0.999
4H-Benzo[def]carbazole	192.0813293	203-65-6	3.82	5-800	0.993
Triphenylphosphate	327.078626	115-86-6	4.59	0.1-800	1.000
Oxybenzone	229.0864742	131-57-7	3.79	0.1-800	0.997
5-nitroacenaphthene	200.0711585	602-87-9	3.85	0.1-800	0.997

Tributyl phosphate	267.1725263	126-73-8	4	0.1-100	0.998
Tris(2-butoxyethyl) phosphate	399.2511706	78-51-3	3.75	0.1-800	0.999
8,10-Dimethylbenz(a)acridine	258.1282795	53-69-0	5.59	0.1-50	0.992
Dioctyl phenylphosphonate	383.2715121	1754-47-8	7.51	0.1-800	0.992
Tris(p-tert-butylphenyl) phosphate	495.2664267	78-33-1	10.43	0.1-800	0.996
Tris(2-ethylhexyl) phosphate	435.3603271	78-42-2	9.49	0.1-800	0.995
Irganox 1076	548.5042958	2082-79-3	13.41	0.1-500	0.999
LC-HRMS NEG					
2,4,4'-Trihydroxybenzophenone	229.0500788	1470-79-7	2.48	0.1-800	0.999
bis(2,4-dihydroxyphenyl)methanone	245.0449934	131-55-5	2.78	0.1-800	0.999
2,4-Dihydroxybenzophenone	213.0551642	131-56-6	2.96	0.1-800	0.999
4-tert-butylphenol	149.0966351	98-54-4	3.31	0.5-100	0.991
Phenol, 2,6-bis(1,1-dimethylethyl)-	205.1592353	128-39-2	4.92	0.1-800	0.997
2,6-ditert-butyl-4-methylphenol	219.1748854	128-37-0	5.1	0.1-800	0.995
2,4-ditert-butylphenol	205.1592353	96-76-4	5.19	0..1-800	0.997
7H-Dibenzo[c,g]carbazole	266.0969694	194-59-2	5.58	0.1-800	0.991
2,4,6-tritert-butylphenol	261.2218356	732-26-3	6.06	0.1-800	0.994
2,2'-Methylenebis(4-methyl-6-tert-butylphenol)	339.2324003	119-47-1	6.25	0.1-800	0.996
2,2'-Methylenebis(6-tert-butyl-4-ethylphenol)	367.2637004	88-24-4	8.95	0.1-800	0.998
4,4'-Methylenebis(2,6-DI-tert-butylphenol)	423.3263006	118-82-1	8.99	0.1-800	0.998
2,2'-Ethylidenebis(4,6-di-tert-butylphenol)	437.3419507	35958-30-6	9.13	0.5-800	0.995
Irganox 1076	529.4620658	2082-79-3	13.41	0.1-500	0.996
Irganox 330	773.5872664	1709-70-2	17.17	0.5-800	0.998
Irganox 1010	1175.776249	6683-19-8	23	0.1-800	0.997
4-nitro-1H-indole	161.0350974	4769-97-5	na	0.1-800	0.995
3,6-Dimethyl-9H-carbazole	194.0969694	5599-50-8	na	0.1-500	0.994
2,4,4'-Trihydroxybenzophenone	229.0500788	1470-79-7	2.48	0.1-800	0.999
2,4-Dihydroxybenzophenone	213.0551642	131-56-6	2.96	0.1-800	0.999
Irganox 1310	277.1803647	20170-32-5	2.9	0.1-800	0.997
Phenol, 2,6-bis(1,1-dimethylethyl)-	205.1592353	128-39-2	4.92	0.1-800	0.997
Irganox 1076	529.4620658	2082-79-3	13.41	0.1-500	0.996

Table S7. Chemical features detected in non-targeted analysis using GCxGC/ToF-MS and LC-HRMS, and their estimated concentrations (minimum, maximum, mean). (ID = Level of confidence in identification, Score % = NIST or mzCloud score, DF = detection frequency).

IUPAC Name	Formula	Exact mass	CAS	Score (%)	ID	Method/ ESI adduct	DF (%)	Concentration (ng/g)		
								Min	Max	Mean
2-methylaniline	C7H9N	107.0735	95-53-4	95	2	2D GC	71	0	65	6
phenylmethanol	C7H8O	108.0575	100-51-6	91	2	2D GC	90	0	7	2
azepan-2-one	C6H11NO	113.0841	105-60-2	96	2	2D GC	95	0	117	9
5H-cyclopenta[b]pyridine	C8H7N	117.0578	120-72-9	94	1	2D GC	71	0	4	1
2-aminobenzonitrile	C7H6N2	118.0531	1885-29-6	90	2	2D GC	29	0	6	1
1-phenylethanone	C8H8O	120.0575	98-86-2	95	2	2D GC	95	0	69	7
1,2,3-trimethylbenzene	C9H12	120.0939	526-73-8	91	2	2D GC	67	0	6	1
2-phenylethanol	C8H10O	122.0732	60-12-8	93	2	2D GC	71	0	7	1
benzoic acid	C7H6O2	122.0368	65-85-0	96	2	2D GC	86	0	270	39
2-methoxyaniline	C7H9NO	123.0684	90-04-0	97	2	2D GC	76	0	40	3
2-chloroaniline	C6H6ClN	127.0189	95-51-2	91	2	2D GC	76	0	3	0
naphthalene	C10H8	128.0626	91-20-3	93	1	2D GC	86	0	21	5
1-isocyanato-2-methylbenzene	C8H7NO	133.0528	614-68-6	93	2	2D GC	48	0	38	3
benzene-1,3-dicarbaldehyde	C8H6O2	134.0368	626-19-7	95	2	2D GC	10	0	12	1
2,4-dimethylbenzaldehyde	C9H10O	134.0732	15764-16-6	94	2	2D GC	95	0	70	13
1,2,4,5-tetramethylbenzene	C10H14	134.1096	95-93-2	89	2	2D GC	90	0	20	4
1,3-benzothiazole	C7H5NS	135.0143	95-16-9	91	1	2D GC	76	0	2	1
N-(2-methylphenyl)formamide	C8H9NO	135.0684	94-69-9	89	2	2D GC	38	0	10	1
4-methoxybenzaldehyde	C8H8O2	136.0524	123-11-5	93	2	2D GC	71	0	5	1
2-aminobenzamide	C7H8N2O	136.0637	88-68-6	89	2	2D GC	14	0	5	0
2-phenoxyethanol	C8H10O2	138.0681	122-99-6	94	2	2D GC	95	0	46	13
1-methylnaphthalene	C11H10	142.0783	90-12-0	94	2	2D GC	95	0	15	4
1-methylnaphthalene	C11H10	142.0783	90-12-0	94	2	2D GC	95	0	11	3
chromen-2-one	C9H6O2	146.0368	91-64-5	95	2	2D GC	81	0	19	2
3H-quinazolin-4-one	C8H6N2O	146.0480	491-36-1	90	2	2D GC	24	0	5	1
isoindole-1,3-dione	C8H5NO2	147.0320	85-41-6	95	2	2D GC	76	0	7	2
4-propylbenzaldehyde	C10H12O	148.0888	28785-06-0	94	2	2D GC	100	1	59	13
3-methylbutan-2-ylbenzene	C11H16	148.1252	4481-30-5	87	2	2D GC	90	0	381	56
N-(2-methylphenyl)acetamide	C9H11NO	149.0841	120-66-1	88	2	2D GC	24	0	3	0

N-(2,4-dimethylphenyl)formamide	C9H11NO	149.0841	60397-77-5	89	2	2D GC	33	0	6	1
1,3-benzodioxole-5-carbaldehyde	C8H6O3	150.0317	120-57-0	91	2	2D GC	81	0	3	1
methyl 2-aminobenzoate	C8H9NO2	151.0633	134-20-3	96	2	2D GC	90	0	14	2
methyl 2-hydroxybenzoate	C8H8O3	152.0473	119-36-8	96	2	2D GC	100	0	384	33
1-chloro-2-isocyanatobenzene	C7H4ClNO	152.9981	3320-83-0	90	2	2D GC	38	0	6	0
(2S,5S)-5-methyl-2-propan-2-ylcyclohexan-1-one	C10H18O	154.1358	491-07-6	88	2	2D GC	71	0	11	2
1,1'-biphenyl	C12H10	154.0783	92-52-4	89	2	2D GC	95	0	2	1
4-chloro-3,5-dimethylphenol	C8H9ClO	156.0342	88-04-0	88	2	2D GC	62	0	7	1
1,5-dimethylnaphthalene	C12H12	156.0939	575-37-1	92	1	2D GC	95	0	6	2
2,6-dimethylnaphthalene	C12H12	156.0939	581-42-0	93	1	2D GC	95	0	14	3
2-methoxynaphthalene	C11H10O	158.0732	93-04-9	93	2	2D GC	100	1	691	94
2,5-dichloroaniline	C6H5Cl2N	160.9799	95-82-9	94	2	2D GC	90	0	11	1
4-tert-butylbenzaldehyde	C11H14O	162.1045	939-97-9	92	2	2D GC	81	0	27	4
4-(2-methylbutan-2-yl)phenol	C11H16O	164.1201	80-46-6	93	2	2D GC	95	0	128	11
4-(2-methylbutan-2-yl)phenol	C11H16O	164.1201	80-46-6	93	2	2D GC	76	0	435	36
phthalic acid	C8H6O4	166.0266	88-99-3	94	2	2D GC	100	0	8	1
9H-fluorene	C13H10	166.0783	86-73-7	88	1	2D GC	95	0	2	1
1-methyl-4-phenylbenzene	C13H12	168.0939	644-08-6	88	2	2D GC	95	0	6	2
1-naphthalen-2-ylethanone	C12H10O	170.0732	93-08-3	95	2	2D GC	90	0	53	5
1,4,6-trimethylnaphthalene	C13H14	170.1096	2131-42-2	94	2	2D GC	95	0	12	3
phenoxybenzene	C12H10O	170.0732	101-84-8	89	2	2D GC	100	0	165	23
2-phenylphenol	C12H10O	170.0732	90-43-7	83	2	2D GC	57	0	1	0
2-ethoxynaphthalene	C12H12O	172.0888	93-18-5	94	2	2D GC	86	0	69	9
phenanthrene	C14H10	178.0783	85-01-8	93	1	2D GC	95	0	15	5
propyl 4-hydroxybenzoate	C10H12O3	180.0786	94-13-3	90	2	2D GC	38	0	6	1
2-hydroxyethyl 4-methylbenzoate	C10H12O3	180.0786	28129-15-9	92	2	2D GC	38	0	14	3
2-methylsulfanyl-1,3-benzothiazole	C8H7NS2	181.0020	615-22-5	86	2	2D GC	52	0	1	0
1-methyl-4-(4-methylphenyl)benzene	C14H14	182.1096	613-33-2	87	2	2D GC	81	0	2	1
1-ethyl-2-phenylbenzene	C14H14	182.1096	1812-51-7	88	2	2D GC	52	0	4	0
diphenylmethanone	C13H10O	182.0732	119-61-9	95	2	2D GC	100	0	514	139
N,N-diethyl-3-methylbenzamide	C12H17NO	191.1310	134-62-3	93	1	2D GC	95	0	156	30
1-methylphenanthrene	C15H12	192.0939	832-69-9	91	1	2D GC	71	0	5	1
1-methylphenanthrene	C15H12	192.0939	832-69-9	92	2	2D GC	95	0	5	1
ethyl 4-(dimethylamino)benzoate	C11H15NO2	193.1103	10287-53-3	91	2	2D GC	86	0	2	1
dimethyl benzene-1,2-dicarboxylate	C10H10O4	194.0579	131-11-3	94	1	2D GC	95	0	415	22
1,3,7-trimethylpurine-2,6-dione	C8H10N4O2	194.0804	58-08-2	95	2	2D GC	76	0	62	12

ethyl 4-ethoxybenzoate	C11H14O3	194.0943	23676-09-7	93	2	2D GC	100	0	216	31
3-phenylpropylbenzene	C15H16	196.1252	1081-75-0	93	2	2D GC	90	0	401	23
fluoranthene	C16H10	202.0783	206-44-0	92	2	2D GC	76	0	5	1
Pyrene	C16H10	202.0783	129-00-0	94	2	2D GC	76	0	18	2
2-benzylideneheptanal	C14H18O	202.1358	122-40-7	91	2	2D GC	86	0	50	7
2-butylisoindole-1,3-dione	C12H13NO2	203.0946	1515-72-6	90	2	2D GC	67	0	9	1
9-ethenylanthracene	C16H12	204.0939	2444-68-0	92	2	2D GC	67	0	26	1
3-(4-tert-butylphenyl)-2-methylpropanal	C14H20O	204.1514	80-54-6	88	2	2D GC	86	0	66	9
4-phenyl-1,2-dihydronaphthalene	C16H14	206.1096	7469-40-1	91	2	2D GC	57	0	63	3
2,6-ditert-butylphenol	C14H22O	206.1671	128-39-2	87	1	2D GC	95	0	9	2
pentyl 2-hydroxybenzoate	C12H16O3	208.1099	2050-08-0	92	2	2D GC	95	0	152	20
pentyl 2-hydroxybenzoate	C12H16O3	208.1099	2050-08-0	90	2	2D GC	95	0	326	40
1,3-diphenylpropan-1-one	C15H14O	210.1045	1083-30-3	89	2	2D GC	81	0	15	1
benzyl benzoate	C14H12O2	212.0837	120-51-4	95	1	2D GC	95	0	841	123
methyl dodecanoate	C13H26O2	214.1933	111-82-0	91	2	2D GC	90	0	112	16
6-chloro-4-N-ethyl-2-N-propan-2-yl-1,3,5-triazine-2,4-diamine	C8H14ClN5	215.0938	1912-24-9	91	2	2D GC	10	0	19	1
2-benzylideneoctanal	C15H20O	216.1514	101-86-0	94	2	2D GC	90	0	281	57
benzenesulfonylbenzene	C12H10O2S	218.0402	127-63-9	93	2	2D GC	95	0	393	43
decan-4-ylbenzene	C16H26	218.2035	4537-12-6	90	2	2D GC	86	0	298	42
decan-2-ylbenzene	C16H26	218.2035	4537-13-7	87	2	2D GC	86	0	247	36
cyclopentyl 4-ethylbenzoate	C14H18O2	218.1307	0-00-0	86	2	2D GC	43	0	11	1
(2-methyl-1-phenylpropan-2-yl) butanoate	C14H20O2	220.1463	10094-34-5	90	2	2D GC	90	0	118	22
diethyl benzene-1,2-dicarboxylate	C12H14O4	222.0892	84-66-2	95	1	2D GC	100	2	89	38
4-phenyl-3,4-dihydro-2H-naphthalen-1-one	C16H14O	222.1045	14578-68-8	89	2	2D GC	38	0	55	3
hexyl 2-hydroxybenzoate	C13H18O3	222.1256	6259-76-3	92	2	2D GC	100	0	1033	120
3-amino-1,1-diphenylurea	C13H13N3O	227.1059	603-51-0	87	2	2D GC	86	0	2	0
(2-hydroxy-4-methoxyphenyl)-phenylmethanone	C14H12O3	228.0786	131-57-7	92	1	2D GC	86	0	146	18
4-[2-(4-hydroxyphenyl)propan-2-yl]phenol	C15H16O2	228.1150	80-05-7	88	2	2D GC	100	0	39	10
benzyl 2-hydroxybenzoate	C14H12O3	228.0786	118-58-1	94	2	2D GC	95	0	716	105
dipropan-2-yl hexanedioate	C12H22O4	230.1518	6938-94-9	93	2	2D GC	81	0	15	2
undecan-4-ylbenzene	C17H28	232.2191	4536-86-1	90	2	2D GC	86	0	321	46
2,4-bis(2-methylbutan-2-yl)phenol	C16H26O	234.1984	120-95-6	91	2	2D GC	71	0	80	11
1-methyl-7-propan-2-ylphenanthrene	C18H18	234.1409	483-65-8	88	1	2D GC	95	0	3	1
	C15H22O2	234.1620	1620-98-0	90	2	2D GC	95	0	58	15

4-phenyl-1,2-di(propan-2-yl)benzene	C18H22	238.1722	61434-46-6	93	2	2D GC	62	0	17	1
methyl 2-benzoylbenzoate	C15H12O3	240.0786	606-28-0	90	2	2D GC	95	0	65	12
methyl tetradecanoate	C15H30O2	242.2246	124-10-7	91	2	2D GC	90	0	166	22
dodecan-6-ylbenzene	C18H30	246.2348	2719-63-3	91	2	2D GC	95	0	515	89
dodecan-2-ylbenzene	C18H30	246.2348	2719-61-1	88	2	2D GC	95	0	354	57
1-(3,5-ditert-butyl-4-hydroxyphenyl)ethanone	C16H24O2	248.1776	14035-33-7	91	2	2D GC	95	0	24	6
2-ethylhexyl 2-hydroxybenzoate	C15H22O3	250.1569	118-60-5	85	2	2D GC	100	0	420	86
bis(2-hydroxyethyl) benzene-1,4-dicarboxylate	C12H14O6	254.0790	959-26-2	86	2	2D GC	86	0	429	93
2,2-dimethoxy-1,2-diphenylethanone	C16H16O3	256.1099	24650-42-8	93	2	2D GC	90	0	72	15
phenyl-(4-phenylphenyl)methanone	C19H14O	258.1045	2128-93-0	92	2	2D GC	67	0	16	5
(3,3,5-trimethylcyclohexyl) 2-hydroxybenzoate	C16H22O3	262.1569	118-56-9	90	2	2D GC	86	0	46	8
(3,3,5-trimethylcyclohexyl) 2-hydroxybenzoate	C16H22O3	262.1569	118-56-9	94	2	2D GC	90	0	305	65
methyl hexadecanoate	C17H34O2	270.2559	112-39-0	89	2	2D GC	95	0	430	105
propan-2-yl tetradecanoate	C17H34O2	270.2559	110-27-0	90	2	2D GC	100	0	170	46
dibutyl benzene-1,2-dicarboxylate	C16H22O4	278.1518	84-74-2	95	1	2D GC	100	3	306	78
bis(2-methylpropyl) benzene-1,2-dicarboxylate	C16H22O4	278.1518	84-69-5	92	2	2D GC	100	1	170	40
2-chloro-N-(2-ethyl-6-methylphenyl)-N-(1-methoxypropan-2-yl)acetamide	C15H22ClNO2	283.1339	51218-45-2	87	2	2D GC	19	0	5	0
ethyl hexadecanoate	C18H36O2	284.2715	628-97-7	88	2	2D GC	95	0	214	42
2-ethylhexyl 3-(4-methoxyphenyl)prop-2-enoate	C18H26O3	290.1882	5466-77-3	90	2	2D GC	86	0	10	3
2-ethylhexyl 3-(4-methoxyphenyl)prop-2-enoate	C18H26O3	290.1882	5466-77-3	95	2	2D GC	95	0	141	30
tridecyl benzoate	C20H32O2	304.2402	29376-83-8	92	2	2D GC	76	0	59	13
2-O-benzyl 1-O-butyl benzene-1,2-dicarboxylate	C19H20O4	312.1362	85-68-7	95	1	2D GC	86	0	26	6
methyl (1R,4aR,4bS,10aR)-1,4a-dimethyl-7-propan-2-yl-2,3,4,4b,5,9,10,10a-octahydrophenanthrene-1-carboxylate	C21H30O2	314.2246	1235-74-1	89	2	2D GC	95	0	65	11
2,4-bis(2-phenylpropan-2-yl)phenol	C24H26O	330.1984	2772-45-4	88	2	2D GC	90	0	141	21
dicyclohexyl benzene-1,2-dicarboxylate	C20H26O4	330.1831	84-61-7	93	1	2D GC	62	0	165	18
diethoxy-sulfanylidene-(3,5,6-trichloropyridin-2-yl)oxy-λ5-phosphane	C9H11Cl3NO3PS	348.9263	2921-88-2	89	2	2D GC	24	0	4	0

tributyl 2-acetyloxypropane-1,2,3-tricarboxylate	C20H3O8	402.2254	77-90-7	93	2	2D GC	95	0	183	23
2,2,6,6-Tetramethylpiperidin-4-ol	C9H19NO	157.1468	2403-88-5	95	2	[M+H]	43	0	0	0
2-Aminobenzamide	C7H8N2O	136.0639	88-68-6	88	2	[M+H]	38	0	0	0
3-[(2S)-1-Methylpyrrolidin-2-yl]pyridine	C10H14N2	162.1159	54-11-5	85	2	[M+H]	38	0	0	0
2-[2-[2-[2-(2-hydroxyethoxy)ethoxy]ethoxy]ethoxy]ethanol	C10H22O6	238.1416	4792-15-8	98	2	[M+H]	76	0	1	0
2-[2-[2-[2-[2-(2-hydroxyethoxy)ethoxy]ethoxy]ethoxy]ethoxy]ethanol	C12H26O7	282.1681	2615-15-8	97	2	[M+H]	81	0	1	0
2-[2-[2-[2-[2-(2-hydroxyethoxy)ethoxy]ethoxy]ethoxy]ethoxy]ethanol	C12H26O7	282.1681	2615-15-8	92	2	[M+H]	81	0	1	0
1,3,7-Trimethylpurine-2,6-dione	C8H10N4O2	194.0806	58-08-2	98	2	[M+H]	76	0	0	0
quinolin-8-ol	C9H7NO	145.0532	148-24-3	80	2	[M+H]	10	0	6	0
2-[2-[2-[2-[2-[2-(2-hydroxyethoxy)ethoxy]ethoxy]ethoxy]ethoxy]ethoxy]ethanol	C14H30O8	326.1942	5617-32-3	96	2	[M+H]	76	0	1	0
2-[2-[2-[2-[2-[2-[2-(2-hydroxyethoxy)ethoxy]ethoxy]ethoxy]ethoxy]ethoxy]ethoxy]ethanol	C16H34O9	370.2206	5117-19-1	97	2	[M+H]	67	0	1	0
3-Methyl-1H-quinoxalin-2-one	C9H8N2O	160.0639	14003-34-0	82	2	[M+H]	57	0	7	1
6-phenyl-1,3,5-triazine-2,4-diamine	C9H9N5	187.0861	91-76-9	95	2	[M+H]	52	0	6	1
Chromen-2-one	C9H6O2	146.0371	91-64-5	93	2	[M+H]	90	0	1	0
4-Ethylbenzaldehyde	C9H10O	134.0733	4748-78-1	85	2	[M+H]	10	0	0	0
4-Phenylbutanoic acid	C10H12O2	164.0843	1821-12-1	86	2	[M+H]	5	0	0	0
2-[2-[2-(2-hydroxypropoxy)propoxy]propoxy]propan-1-ol	C12H26O5	250.1783	24800-25-7	88	2	[M+H]	71	0	1	0
N-Ethyl-N-propan-2-ylpropan-2-amine	C8H19N	129.1520	7087-68-5	82	2	[M+H]	5	0	0	0
4-[(4-aminophenyl)methyl]aniline	C13H14N2	198.1161	101-77-9	89	2	[M+H]	24	0	4	0
4-(ethylamino)-6-(propan-2-ylamino)-1H-1,3,5-triazin-2-one	C8H15N5O	197.1281	2163-68-0	97	2	[M+H]	14	0	27	1
N-(2,4-dimethylphenyl)formamide	C9H11NO	149.0845	60397-77-5	91	2	[M+H]	62	0	0	0
2-[2-(2-butoxyethoxy)ethoxy]ethanol	C10H22O4	206.1520	143-22-6	94	2	[M+H]	71	0	1	0
N-ethyl-4-methylbenzenesulfonamide	C9H13NO2S	199.0668	80-39-7	90	2	[M+H]	90	0	0	0
2,6-Dimethylaniline	C8H11N	121.0895	87-62-7	96	2	[M+H]	24	0	0	0

triethyl 2-hydroxypropane-1,2,3-tricarboxylate	C12H20O7	276.1215	77-93-0	82	2	[M+H]	48	0	0	0
(2-hydroxy-4-methoxyphenyl)-phenylmethanone	C14H12O3	228.0789	131-57-7	94	1	[M+H]/[M-H]	76	0	79	5
(2-hydroxy-4-octoxyphenyl)-phenylmethanone	C21H26O3	326.1880	1843-05-6	94	1	[M+H]/[M-H]	76	0	4	0
Penta(propylene glycol)	C15H32O6	308.2203	00-00-00	89	2	[M+H]	71	0	0	0
6,7-dimethoxychromen-2-one	C11H10O4	206.0586	120-08-1	81	2	[M+H]	52	0	7	1
N-butylbenzenesulfonamide	C10H15NO2S	213.0828	3622-84-2	93	2	[M+H]	5	0	0	0
dimethyl benzene-1,4-dicarboxylate	C10H10O4	194.0582	120-61-6	88	2	[M+H]	5	0	2	0
6-chloro-4-N-ethyl-2-N-propan-2-yl-1,3,5-triazine-2,4-diamine	C8H14ClN5	215.0941	1912-24-9	97	2	[M+H]	76	0	5	0
N,N-diethyl-3-methylbenzamide	C12H17NO	191.1311	134-62-3	96	1	[M+H]	90	0	2	0
1,3-bis(4-methylphenyl)urea	C15H16N2O	240.1267	621-00-1	83	2	[M+H]	24	0	0	0
4-(4-amino-3-chlorophenyl)-2-chloroaniline	C12H10Cl2N2	252.0226	91-94-1	90	2	[M+H]	19	0	1	0
Acridine	C13H9N	179.0740	260-94-6	81	2	[M+H]	76	0	1	0
benzyl (E)-3-phenylprop-2-enoate	C16H14O2	238.0995	103-41-3	83	2	[M+H]	14	0	0	0
2-[2-[2-[2-[2-[2-(2-hydroxypropoxy)propoxy]propoxy]propoxy]propoxy]propoxy]propan-1-ol	C21H44O8	424.3038	14362-16-4	88	2	[M+H]	57	0	0	0
(2*Z*,4*S*,5*S*,6*S*,7*R*,8*Z*)-2,9-Diphenyldeca-2,8-diene-3,4,5,6,7,8-hexol	C22H26O6	386.1730	00-00-00	93	2	[M+H]	100	0	161	23
Diphenylmethanone	C13H10O	182.0735	119-61-9	93	2	[M+H]	100	0	853	108
11H-benzo[b][1]benzazepine	C14H11N	193.0898	256-96-2	87	2	[M+H]	38	0	0	0
(2E,4E)-5-(1,3-benzodioxol-5-yl)-1-piperidin-1-ylpenta-2,4-dien-1-one	C17H19NO3	285.1368	94-62-2	96	2	[M+H]	76	0	32	5
dipropyl hexanedioate	C12H22O4	230.1521	106-19-4	92	2	[M+H]	100	0	24	3
Ethyl 4-ethoxybenzoate	C11H14O3	194.0946	23676-09-7	94	2	[M+H]	48	0	1	0
(3-methylphenyl)-phenylmethanone	C14H12O	196.0893	643-65-2	85	2	[M+H]	100	0	4	1
1-[2,6-Bis(4-ethylphenyl)-4,4a,8,8a-tetrahydro-[1,3]dioxino[5,4-d][1,3]dioxin-4-yl]ethane-1,2-diol	C24H30O6	414.2042	00-00-00	93	2	[M+H]	100	0	525	124
1,3-diphenylpropan-2-one	C15H14O	210.1047	102-04-5	85	2	[M+H]	100	0	63	4
Sodium;2-[dodecanoyl(methyl)amino]acetate	C15H29NO3	271.2157	137-16-6	92	2	[M+H]	14	0	0	0
3,5-ditert-butyl-4-hydroxybenzaldehyde	C15H22O2	234.1622	1620-98-0	89	2	[M+H]	76	0	1	0
bis(2-methylpropyl) benzene-1,2-dicarboxylate	C16H22O4	278.1521	84-69-5	88	2	[M+H]	71	0	1	0

dibutyl hydrogen phosphate	C8H19O4P	210.1023	107-66-4	80	2	[M+H]	10	0	0	0
4,6,6,7,8,8-hexamethyl-4,7-dihydro-3H-cyclopenta[g]isochromen-1-one	C18H24O2	272.1778	256393-37-0	88	2	[M+H]	90	0	0	0
Phenyl-(4-phenylphenyl)methanone	C19H14O	258.1049	2128-93-0	89	2	[M+H]	71	0	0	0
4-[[4-(dimethylamino)phenyl]-phenylmethyl]-N,N-dimethylaniline	C23H26N2	330.2099	129-73-7	81	2	[M+H]	100	0	0	0
(10E,12E)-9-oxooctadeca-10,12-dienoic acid	C18H30O3	294.2199	54232-58-5	96	2	[M+H]	100	0	0	0
bis[4-(diethylamino)phenyl]methanone	C21H28N2O	324.2204	90-93-7	94	2	[M+H]	71	0	0	0
tributyl 2-hydroxy-4-oxopentane-1,2,3-tricarboxylate	C20H34O8	402.2255	77-90-7	95	2	[M+H]	67	0	0	0
dicyclohexyl benzene-1,2-dicarboxylate	C20H26O4	330.1834	84-61-7	86	1	[M+H]	100	0	0	0
4-[[4-(dimethylamino)phenyl]-(4-methyliminocyclohexa-2,5-dien-1-ylidene)methyl]-N,N-dimethylaniline;hydrochloride	C24H27N3	357.2208	603-47-4	91	2	[M+H]	43	0	2	0
	C21H18N4O2	358.1430		89	2	[M+H]	24	0	3	0
methyl (9Z,11E,13E)-octadeca-9,11,13-trienoate	C19H32O2	292.2404	4175-47-7	95	2	[M+H]	57	0	0	0
dibutyl decanedioate	C18H34O4	314.2459	109-43-3	96	2	[M+H]	62	0	0	0
Hexadecanamide	C16H33NO	255.2563	629-54-9	92	2	[M+H]	100	0	0	0
1,3-bis[3-(4,5-dihydro-1H-imidazol-2-yl)phenyl]urea	C19H20N6O	348.1699	27885-92-3	89	2	[M+H]	33	0	2	0
methyl (Z)-octadec-12-enoate	C19H36O2	296.2718	2733-86-0	93	2	[M+H]	62	0	2	0
octadecanamide	C18H37NO	283.2877	124-26-5	93	2	[M+H]	71	0	2	0
bis(2-ethylhexyl) hexanedioate	C22H42O4	370.3085	103-23-1	96	2	[M+H]	52	0	4	0
(2S)-1,2,3-trihydroxyhenicosan-4-one	C21H42O4	358.3084	00-00-00	89	2	[M+H]	81	0	3	0
[2,2-bis[3-(3,5-ditert-butyl-4-hydroxyphenyl)propanoyloxymethyl]-3-hydroxypropyl] 3-(3,5-ditert-butyl-4-hydroxyphenyl)propanoate	C56H84O10	916.6056	84633-54-5	0	2	[M+NH4+]	62	0	0	0
tetradecan-1-amine	C14H31N	213.2459	2016-42-4	89	2	[M+H]	100	0	1	0
(Z)-docos-13-enamide	C22H43NO	337.3346	112-84-5	92	2	[M+H]	76	0	7	2
2-(2H-Benzotriazol-2-yl)-4,6-bis(1-methyl-1-phenylethyl)phenol	C30H29N3O	447.2318	70321-86-7	83	2	[M+H]	24	0	0	0
5-tert-butyl-2-[5-(5-tert-butyl-1,3-benzoxazol-2-yl)thiophen-2-yl]-1,3-benzoxazole	C26H26N2O2S	430.1721	7128-64-5	84	2	[M+H]	67	0	0	0
ethyl (*Z*)-octadec-9-enoate	C20H38O2	310.2876	111-62-6	87	2	[M+H]	48	0	0	0

didecyl benzene-1,2-dicarboxylate	C28H46O4	446.3390	84-77-5	90	2	[M+H]	62	0	0	0
2-ethyl-N-(2-ethylhexyl)hexan-1-amine	C16H35N	241.2772	106-20-7		2	[M+H]	62	0	42	4
docosanamide	C22H45NO	339.3503	3061-75-4	96	2	[M+H]	86	0	1	0
4-[[4,6-bis(octylsulfanyl)-1,3,5-triazin-2-yl]amino]-2,6-ditert-butylphenol	C33H56N4OS2	588.3893	991-84-4	91	2	[M+H]	76	0	32	2
tris(2,4-ditert-butylphenyl) phosphate	C42H63O4P	662.4462	95906-11-9	0	1	[M+H]	100	0	1	1
Octadecan-1-amine	C18H39N	269.3086	124-30-1	99	2	[M+H]	43	0	0	0
Octadecyl 3-(3,5-ditert-butyl-4-hydroxyphenyl)propanoate	C35H62O3	547.4974	2082-79-3	0	1	[M+NH4+]	86	0	76	14
(2-hydroxy-3-octadeca-9,12-dienoyloxypropyl) octadec-9-enoate	C39H70O5	618.5202	104346-53-4	0	2	[M+H]	67	0	25	3
octadecyl (E)-3-(3,5-ditert-butyl-4-hydroxyphenyl)prop-2-enoate	C35H60O3	528.4549	19277-65-7	0	2	[M+H]	100	0	10	2
Tris(2,4-ditert-butylphenyl) phosphite	C42H63O3P	646.4518	31570-04-4	95	1	[M+H]	100	0	3	1
2,4-ditert-butyl-6-(5-chlorobenzotriazol-2-yl)phenol	C20H24ClN3O	357.1608	3864-99-1	83	2	[M-H]	5	0	0	0
4-(4-hydroxyphenyl)sulfonylphenol	C12H10O4S	250.0294	80-09-1	89	2	[M-H]	100	0	0	0
4-[2-(4-hydroxyphenyl)propan-2-yl]phenol	C15H16O2	228.1140	80-05-7	88	2	[M-H]	100	0	39	4
2-[(2S,4aR,8aS)-2-hydroxy-4a-methyl-8-methylidene-3,4,5,6,7,8a-hexahydro-1H-naphthalen-2-yl]prop-2-enoic acid	C15H22O3	250.1565	00-00-00	92	2	[M-H]	100	0	4	0
(Z)-9,12,13-trihydroxyoctadec-15-enoic acid	C18H34O5	330.2397	00-00-00	85	2	[M-H]	100	0	0	0
4-(4-hydroxy-3-prop-2-enylphenyl)sulfonyl-2-prop-2-enylphenol	C18H18O4S	330.0919	41481-66-7	88	2	[M-H]	100	0	0	0
(Z)-docos-13-enoic acid	C22H42O2	338.3178	112-86-7	96	2	[M-H]	57	0	2	0
Dodecyl hydrogen sulfate	C12H26O4S	266.1544	151-41-7	92	2	[M-H]	100	0	22	1
4-[1,1,1,3,3,3-hexafluoro-2-(4-hydroxyphenyl)propan-2-yl]phenol	C15H10F6O2	336.0579	1478-61-1	87	2	[M-H]	5	0	0	0
tridecyl hydrogen sulfate	C13H28O4S	280.1697	68611-55-2		2	[M-H]	14	0	1	0
tetradecyl hydrogen sulfate	C14H30O4S	294.1855	4754-44-3	88	2	[M-H]	100	0	832	40
3-(3,5-ditert-butyl-4-hydroxyphenyl)propanoic acid	C17H26O3	278.1869	20170-32-5		1	[M-H]	100	0	6	0
2,5-ditert-butylbenzene-1,4-diol	C14H22O2	222.1611	88-58-4	83	2	[M-H]	76	0	0	0
4-dodecylbenzenesulfonic acid	C18H30O3S	326.1910	121-65-3	96	2	[M-H]	10	0	10	1
2,6-ditert-butylphenol	C14H22O	206.1660	128-39-2	82	1	[M-H]	95	0	14	4
NA	C14H24O2	224.1766	82909-47-5	89	2	[M-H]	62	0	1	0

16-hydroxyhexadecanoic acid	C16H32O3	272.2348	506-13-8	85	2	[M-H]	57	0	1	0
tetradecanoic acid	C14H28O2	228.2080	544-63-8	91	2	[M-H]	62	0	50	8
(2E,4E,6E,8E)-3,7-dimethyl-9-(2,6,6-trimethylcyclohexen-1-yl)nona-2,4,6,8-tetraenoic acid	C20H28O2	300.2081	302-79-4	85	2	[M-H]	62	0	1	0
(*Z*)-octadec-9-en-17-ynoic acid	C18H30O2	278.2238	151333-45-8	93	2	[M-H]	43	0	6	0
pentadecanoic acid	C15H30O2	242.2238	1002-84-2	93	2	[M-H]	52	0	1	0
2,4-bis(2-methylbutan-2-yl)phenol	C16H26O	234.1974	120-95-6	93	2	[M-H]	71	0	0	0
2,4-bis(2-phenylpropan-2-yl)phenol	C24H26O	330.1977	2772-45-4	97	2	[M-H]	86	0	1	0
(Z)-hexadec-9-enoic acid	C16H30O2	254.2239	373-49-9	98	2	[M-H]	67	0	2	0
(9Z,12Z)-octadeca-9,12-dienoic acid	C18H32O2	280.2394	60-33-3	94	2	[M-H]	48	0	20	2
1-Phenanthrenecarboxylic acid, 1,2,3,4,4a,4b,5,6,10,10a-decahydro-1,4a-dimethyl-7-(1-methylethyl)-, (1R,4aR,4bR,10aR)-	C20H30O2	302.2238	514-10-3	98	2	[M-H]	67	0	1	0
ethyl tetradecanoate	C16H32O2	256.2394	124-06-1	98	2	[M-H]	67	0	15	3
2-[(1S,3R)-3-hydroxycyclohexyl]-5-(2-methylheptan-2-yl)phenol	C20H32O2	304.2395	132296-20-9	97	2	[M-H]	100	0	22	3
methyl (11Z,14Z)-icosa-11,14-dienoate	C20H36O2	308.2707	61012-46-2	91	2	[M-H]	100	0	7	1
octadecanoic acid	C18H36O2	284.2706	57-11-4	96	2	[M-H]	76	0	8	3
(Z)-icos-14-enoic acid	C20H38O2	310.2864	17735-95-4	94	2	[M-H]	52	0	1	0
icosanoic acid	C20H40O2	312.3020	506-30-9	93	2	[M-H]	57	0	1	0
2-tert-butyl-6-(5-chlorobenzotriazol-2-yl)-4-methylphenol	C17H18ClN3O	315.1133	3896-11-5	92	2	[M-H]	100	0	7	0
(Z)-tetracos-15-enoic acid	C24H46O2	366.3489	506-37-6	96	2	[M-H]	100	0	0	0
4-[[3,5-bis[(3,5-ditert-butyl-4-hydroxyphenyl)methyl]-2,4,6-trimethylphenyl]methyl]-2,6-ditert-butylphenol	C54H78O3	774.5944	1709-70-2	91	1	[M-H]	100	0	2	0
pentacosanoic acid	C25H50O2	382.3808	506-38-7	98	2	[M-H]	14	0	0	0
tetracosanoic acid	C24H48O2	368.3644	557-59-5	92	2	[M-H]	29	0	0	0
[3-[3-(3,5-ditert-butyl-4-hydroxyphenyl)propanoyloxy]-2,2-bis[3-(3,5-ditert-butyl-4-hydroxyphenyl)propanoyloxymethyl]propyl] 3-(3,5-ditert-butyl-4-hydroxyphenyl)propanoate	C73H108O12	1176.7841	6683-19-8	90	1	[M+NH4]/[M-H]	76	0	7	2
(Z)-octadec-9-enoic acid	C18H34O2	282.2562	112-80-1	89	2	[M+H]/[M-H]	76	0	14	2

Table S8. Detection frequencies (DFs) and concentrations (minimum (Min), maximum (Max), mean, and standard deviations (SD)) of target organic chemicals in plastic recyclates in ng/g. (DL = Detection Limit)

Analyte	Abbrev.	DF (%)	Min	Max	Mean	SD
Organophosphorus flame retardants (OPFRs)						
Trimethyl phosphate	TMP	86	<DL	139.0	11.19	32.57
Triethyl phosphate	TEP	90	<DL	2.6	0.51	0.72
Tributylphosphine oxide	TBPO	62	<DL	0.4	0.15	0.10
Tributyl phosphate	TNBP	100	0	622.4	34.63	134.78
Triisobutyl phosphate	TIBP	100	0	4.1	1.07	1.19
Triphenylphosphine oxide	TPPO	86	<DL	29.2	5.29	6.96
Tris(2-chloroethyl)phosphate	TCEP	57	<DL	4.4	1.20	1.52
Tris(2-chloroisopropyl) phosphate	TCPP	90	<DL	102.8	28.63	29.35
Tris(1,3-dichloroisopropyl)phosphate	TDCIPP	76	<DL	11.2	2.95	3.58
Triphenyl phosphate	TPHP	90	<DL	117.6	19.25	27.90
2-ethylhexyl diphenyl phosphate	EHDPP	95	<DL	3855.5	396.42	861.90
Tri-m and p-totyl phosphate (tricresyl phosphate isomers)	m,p-TOTP	81	<DL	15.9	4.86	5.35
Isodecyl diphenyl Phosphate	IDDPP	90	<DL	60.4	9.28	18.11
Tris(2-butoxyethyl) phosphate	TBOEP	95	<DL	123.5	26.23	33.85
Tris(2-ethylhexyl)phosphate	TEHP	67	<DL	11.4	3.34	3.62
Bis(p-tert-butylphenyl) phenyl phosphate	DTBPPP	81	<DL	55.3	4.02	13.26
Tris(2-isopropylphenyl)phosphate	T2IPPP	86	<DL	0.4	0.07	0.08
tris(p-tert-Butylphenyl) phosphate	TTBPP	57	<DL	2.6	0.43	0.72
(4-tert-butylphenyl) diphenyl phosphate	TBDPP	67	<DL	282.3	23.86	74.51
Diphenyl-3-isopropylphenyl phosphate	DIPIPP	67	<DL	4.4	0.52	1.14
Tris(xylyl phenyl)phosphate	TXP	43	<DL	0.8	0.30	0.23
Tri-o-totyl phosphate (tricresyl phosphate isomers)	o-TOTP	24	<DL	3.9	2.07	1.48
Tri-propyl phosphate	TPrP	5	<DL	0.2	0.24	na
Tetraethylethylene diphosphonate	TEEDP	5	<DL	0.1	0.05	na
Dioctylphenyl phosphonate	DOPP	5	<DL	0.1	0.06	na
Tris(2,3-dibromopropyl) phosphate	TDBPP	0	<DL	nd	nd	nd
Brominated Flame Retardants (BFRs)						
2-ethyl-1-hexyl 2,3,4,5-tetrabromobenzoate	EHTBB	76	<DL	2147.2	422.00	440.37
Allyl 2,4,6-tribromophenyl ether	ATE	38	0	74.3	27.76	36.27
2,3,5,6-Tetrabromo-p-xylene	P-TBX	33	0	107.3	35.03	50.78
Pentabromoethylbenzene	PBEB	24	0	41.3	9.65	17.70
Hexabromocyclododecane	g-HBCD	33	0	649.0	211.11	306.03
Pentabromophenyl-methyl acrylate	PBBA	33	0	129.4	42.03	60.94
Pentabromobenzene	PBBZ	19	0	64.9	12.02	25.40
Bis(2-ethyl-1-hexyl)tetrabromophthalate	BEHTBP	19	0	405.9	75.66	159.85
2-Bromoallyl 2,4,6-tribromophenyl ether	BATE	5	0	89.8	4.28	19.60
tetrabromo-o-chlorotoluene	TBCT		n.d.	n.d.		
Pentabromotoluene	PBT		n.d.	n.d.		

2,3-Dibromopropyl 2,4,6-tribromophenyl ether	DPTE		n.d.	n.d.		
Hexabromobenzene	HBB		n.d.	n.d.		
2,2',4,5,5'-Pentabromobiphenyl	BB-101		n.d.	n.d.		
1,2-Bis(2,4,6-tribromophenoxy)ethane	BTBPE		n.d.	n.d.		
Octabromotrimethylphenyl indane	O-BIND		n.d.	n.d.		
Syn-Dechlorane Plus	S-DP		n.d.	n.d.		
Anti-Dechlorane Plus	A-DP		n.d.	n.d.		
2,2',4,4',5-Penta	BDE-99	48	0.31	14.3	1.25	3.13
2,2',4,4'-Tetra	BDE-47	24	0.48	14.6	1.05	3.21
2,2',4,4',6-Penta	BDE-100	14	0.48	2.5	0.17	0.56
2,2',3,4,4',5',6-Hepta	BDE-183	19	0.52	2.4	0.28	0.65
2,2',4,4',5,6'-Hexa	BDE-154	14	0.14	0.9	0.08	0.24
2,2',4,4',5,5'-Hexa	BDE-153	14	0.54	1.1	0.10	0.28
2,2',3,4,4',5,6-Hepta	BDE-181	10	0.43	0.6	0.05	0.17
2,3',4,4',5-Penta	BDE-118	5	1.61	1.6	0.08	0.35
3,3',4,4',5-Penta/2,2',4,4',6,6'-Hexa-	BDE-126+155	5	0.67	0.7	0.03	0.15
2,2',3,4,4',5'-/2,3,4,4',5,6-Hexa	BDE-138+166	5	1.90	1.9	0.09	0.42
2,2',3,3',4,4',5,6,6'-Nona	BDE-207	5	2.17	2.2	0.10	0.47
2,2',3,4,4',5,6,6'-/2,2',3,3',4,4',6,6'-Octa	BDE-204+197		n.d.	n.d.		
2,4-Di	BDE-7		n.d.	n.d.		
3,3',4-Tri	BDE-35		n.d.	n.d.		
2,3',4',6-Tetra	BDE-71		n.d.	n.d.		
2,6-Di	BDE-10		n.d.	n.d.		
3,3'-Di	BDE-11+8		n.d.	n.d.		
3,4-/3,4'-Di	BDE-12+13		n.d.	n.d.		
4,4'-Di	BDE-15		n.d.	n.d.		
2,4,6-Tri	BDE-30		n.d.	n.d.		
2,4',6-Tri	BDE-32		n.d.	n.d.		
2,2',4-/2,3',4-Tri	BDE-17+25		n.d.	n.d.		
2,4,4'-/2',3,4-Tri	BDE-28+33		n.d.	n.d.		
3,4,4'-Tri	BDE-37		n.d.	n.d.		
2,4,4',6-Tetra	BDE-75		n.d.	n.d.		
2,2',4,5'-Tetra	BDE-49		n.d.	n.d.		
2,3',4,4'-Tetra	BDE-66		n.d.	n.d.		
3,3',4,4'-Tetra	BDE-77		n.d.	n.d.		
2,3',4,4',6-Penta	BDE-119		n.d.	n.d.		
2,3,4,5,6-Penta	BDE-116		n.d.	n.d.		
2,2',3,4,4'-Penta	BDE-85		n.d.	n.d.		
2,3,3',4,4',5',6-Hepta	BDE-190		n.d.	n.d.		
2,2',3,3',5,5',6,6'-Octa	BDE-202		n.d.	n.d.		
2,2',3,3',4,5',6,6'-Octa	BDE-201		n.d.	n.d.		
2,2',3,3',4,5,5',6'-/ 2,2',3,3',4,5,5',6-/2,2',3,3',4,5,6,6-/ 2,2',3,4,4',5,5',6-octa	BDE-198+199+200+203		n.d.	n.d.		
2,2',3,3',4,4',5,6'-Octa	BDE-196		n.d.	n.d.		

2,2',3,4,4',5,5',6-Octa	BDE-203		n.d.	n.d.		
2,2',3,3',4,4',5,5'-Octa	BDE-194		n.d.	n.d.		
2,2',3,3',4,4',5,6-Octa	BDE-195		n.d.	n.d.		
2,2',3,3',4,5,5',6,6'-Nona	BDE-208		n.d.	n.d.		
2,2',3,3',4,4',5,5',6-Nona	BDE-206		n.d.	n.d.		
Perfluoroalkyl acids (PFAAs)						
Perfluorobutanoic acid	PFBA	5	<DL	0.1	0.01	0.02
Perfluoropentanoic acid	PFPeA	10	<DL	0.1	0.01	0.02
Perfluorohexanoic acid	PFHxA	19	<DL	0.1	0.01	0.03
Perfluoroheptanoic acid	PFHpA	14	<DL	0.0	0.00	0.01
Perfluorooctanoic acid	PFOA	14	<DL	0.0	0.00	0.01
Perfluorononanoic acid	PFNA	10	<DL	0.1	0.01	0.03
Perfluorodecanoic acid	PFDA	10	<DL	0.0	0.00	0.01
Perfluoroundecanoic acid	PFUnA	19	<DL	0.1	0.01	0.02
Perfluorododecanoic acid	*PFDoA*	10	<DL	0.1	0.00	0.01
Perfluorotridecanoic acid	PFTriA	14	<DL	0.0	0.00	0.01
Perfluorotetradecanoic acid	PFTeDA	10	<DL	0.1	0.00	0.02
Perfluorohexadecanoic acid	*PFHxDA*	5	0.01	0.0	0.00	0.00
Perfluorobutanesulfonoic acid	*PFBS*	19	0.02	0.0	0.00	0.01
Perfluorohexanesulfonoic acid	*PFHxS*	10	0.01	0.0	0.00	0.00
Perfluorooctanesulfonoic acid	PFOS	10	<DL	0.0	0.00	0.01
Linear-Perfluorooctanesulfonoic acid	PFOS-L	14	<DL	0.0	0.00	0.01
Perfluorodecanesulfonoic acid	PFDS	5	<DL	0.0	0.00	0.00
Perfluoroheptanesulfonoic acid	*PFHpS*		n.d.	n.d.		
Perfluorooctanesufonamide	PFOSA		n.d.	n.d.		
Perfluoroethylcyclohexane sulfonate	PFECHSb		n.d.	n.d.		
Chloroperfluorooctanesulfonic acid	CL PFOS		n.d.	n.d.		

Table S9. Detection frequencies (DFs) and concentrations (minimum (Min), maximum (Max), mean, and standard deviation (SD)) of elements in plastic recyclates in mg/kg. (DL=Detection Limit; ND = Not Detected)

		DF (%)	Min	Max	Mean	SD
Calcium	Ca	62	<DL	2980	473	894
Sodium	Na	52	<DL	617	68	161
Iron	Fe	48	<DL	156	13	35
Magnesium	Mg	29	<DL	84	11	22
Zinc	Zn	62	<DL	132	8.84	29
Sulfur	S	5	<DL	106	5.58	24
Potassium	K	5	<DL	87	4.58	20
Antimony	Sb	62	<DL	16	3.19	5.33
Phosphorus	P	5	<DL	40	2.00	8.94
Aluminum	Al	5	<DL	39	1.95	8.72
Strontium	Sr	52	<DL	1.46	0.33	0.50
Copper	Cu	24	<DL	2.30	0.25	0.62
Cobalt	Co	48	<DL	0.90	0.20	0.35
Chromium	Cr	62	<DL	1.36	0.16	0.30
Manganese	Mn	10	<DL	2.40	0.15	0.54
Lead	Pb	62	<DL	0.91	0.14	0.24
Barium	Ba	10	<DL	1.70	0.12	0.40
Titanium	Ti	10	<DL	1.00	0.10	0.30
Lithium	Li	33	<DL	0.71	0.09	0.18
Tin	Sn	10	<DL	0.90	0.08	0.26
Mercury	Hg	62	<DL	0.49	0.05	0.11
Nickel	Ni	10	<DL	0.70	0.04	0.16
Bismuth	Bi	24	<DL	0.16	0.01	0.04
Yttrium	Y	29	<DL	0.18	0.01	0.04
Molybdenum	Mo	19	<DL	0.08	0.01	0.02
Cerium	Ce	14	<DL	0.08	0.01	0.02
Lanthanum	La	14	<DL	0.06	0.00	0.01
Neodymium	Nd	29	<DL	0.05	0.00	0.01
Germanium	Ge	10	<DL	0.05	0.00	0.01
Rubidium	Rb	5	<DL	0.07	0.00	0.02
Arsenic	As	5	<DL	0.03	0.00	0.01
Silver	Ag	5	<DL	0.03	0.00	0.01
Tellurium	Te	5	<DL	0.02	0.00	0.00
Praseodymium	Pr	5	<DL	0.01	0.00	0.00
Europium	Eu	5	<DL	0.01	0.00	0.00
Gadolinium	Gd	5	<DL	0.01	0.00	0.00
Uranium	U	10	<DL	0.00	0.00	0.00
Beryllium	Be		ND			
Boron	B		ND			
Cadmium	Cd		ND			
Cesium	Cs		ND			
Gallium	Ga		ND			
Hafnium	Hf		ND			

Holmium	Ho	ND
Iridium	Ir	ND
Lutetium	Lu	ND
Niobium	Nb	ND
Palladium	Pd	ND
Platinum	Pt	ND
Rhodium	Rh	ND
Ruthenium	Ru	ND
Samarium	Sm	ND
Scandium	Sc	ND
Selenium	Se	ND
Terbium	Tb	ND
Thallium	Tl	ND
Tungsten	W	ND
Vanadium	V	ND
Zirconium	Zr	ND
Ytterbium	Yb	ND

REFERENCES

(1) Phillips, K. A.; Yau, A.; Favela, K. A.; Isaacs, K. K.; McEachran, A.; Grulke, C.; Richard, A. M.; Williams, A. J.; Sobus, J. R.; Thomas, R. S.; Wambaugh, J. F. Suspect Screening Analysis of Chemicals in Consumer Products. *Environ. Sci. Technol.* **2018**, *52* (5), 3125–3135. https://doi.org/10.1021/acs.est.7b04781.

(2) Lescord, G. L.; Kidd, K. A.; De Silva, A. O.; Williamson, M.; Spencer, C.; Wang, X.; Muir, D. C. G. Perfluorinated and Polyfluorinated Compounds in Lake Food Webs from the Canadian High Arctic. *Environ. Sci. Technol.* **2015**, *49* (5), 2694–2702. https://doi.org/10.1021/es5048649.

(3) Schymanski, E. L.; Jeon, J.; Gulde, R.; Fenner, K.; Ruff, M.; Singer, H. P.; Hollender, J. Identifying Small Molecules via High Resolution Mass Spectrometry: Communicating Confidence. *Environ. Sci. Technol.* **2014**, *48* (4), 2097–2098. https://doi.org/10.1021/es5002105.

(4) Wang, Y.; Gao, X.; Liu, B.; Lin, Q.; Xia, Y. Identification of Chemicals in a Polyvinyl Chloride/Polyethylene Multilayer Film by Ultra-High-Performance Liquid Chromatography/Quadrupole Time-of-Flight Mass Spectrometry and Their Migration into Solution. *Journal of Chromatography A* **2020**, *1625*, 461274. https://doi.org/10.1016/j.chroma.2020.461274.
